# A Reporting Guideline for Observational Studies in Metabolomic Epidemiology: Explanation and Elaboration of the Strobe-MetEpi Checklist

**DOI:** 10.21203/rs.3.rs-9928764/v1

**Published:** 2026-06-25

**Authors:** Rachel S Kelly, Stacey N Reinke, Georgia Lorentzen, Anastasia Chrysovalantou Chatziioannou, Ruey Leng Loo, Lorraine Brennan, Majken K. Jensen, Elena Colicino, Robert van Vorstenbosch, Amir Hossein Alizadeh Bahmani, Antonio Checa, Burcu F. Darst, Hector Gallart-Ayala, Shu-Yi Liao, Salman Siddiqui, Maria A. Valdivia-Garcia, Anisha Wijeyesekera, David Broadhurst, Steven C. Moore, Timothy Ebbels, Matthias Egger, Royston Goodacre, Julian Little, Craig E. Wheelock, Jessica Lasky-Su

**Affiliations:** 1. Channing Division of Network Medicine, Brigham and Women’s Hospital and Harvard Medical School, Boston, USA; 2. School of Science, Edith Cowan University, 270 Joondalup Drive, Joondalup WA, 6027, Australia; 3. Division of Systems Medicine, Department of Metabolism, Digestion, & Reproduction, Imperial College London, London, United Kingdom; 4. Nutrition and Metabolism Branch, International Agency for Research on Cancer, Lyon, France; 5. Centre for Computational and Systems Medicine, Health Futures Institute, Murdoch University, 5 Robin Warren Drive, Perth, WA 6150 Australia; 6. UCD School of Agriculture and Food Science, Institute of Food and Health, University College Dublin, Dublin 4, Ireland; 7. Department of Public Health, University of Copenhagen, Copenhagen, Denmark; 8. Department of Environmental Medicine, Division of Biostatistics, Icahn School of Medicine at Mount Sinai, New York, NY, USA; 9. Department of Biosciences, University of Milan, Italy; 10. Institute of Risk Assessment Sciences, Utrecht University, Utrecht, The Netherlands; 11. Department of Vascular Medicine, Amsterdam UMC location University of Amsterdam, Meibergdreef 9, 1105 AZ Amsterdam, The Netherlands; 12. Unit of Integrative Metabolomics, Institute of Environmental Medicine, Karolinska Institutet, Stockholm, Sweden; 13. Public Health Sciences, Fred Hutchinson Cancer Center, Seattle, WA, USA 98109; 14. Metabolomics and Lipidomics Facility, University of Lausanne, Switzerland.; 15. Division of Pulmonary and Critical Care Medicine, University of California, Los Angeles; 16. The National Phenome Centre, Section of Bioanalytical Chemistry, Division of Systems Medicine, Department of Metabolism, Digestion and Reproduction, Faculty of Medicine, Imperial College London, Institute of Reproductive and Developmental Biology, Hammersmith Hospital Campus, London W12 0NN, United Kingdom.; 17. National Heart and Lung Institute, Imperial College London, London, UK.; 18. Food Microbial Sciences Unit, Department of Food and Nutritional Sciences, University of Reading, Reading, RG6 6DZ, United Kingdom.; 19. Metabolic Epidemiology Branch, Division of Cancer Epidemiology and Genetics, National Cancer Institute, Rockville, MD, USA.; 20. Section of Bioinformatics, Division of Systems Medicine, Department of Metabolism, Digestion and Reproduction, Imperial College London, Hammersmith Campus, London, W12 0NN, UK; 21. Center for Reproducible Science and Research Synthesis, University of Zurich, Zurich, Switzerland; 22. Institute of Social & Preventive Medicine, University of Bern, Bern, Switzerland; 23. Population Health Sciences, Bristol Medical School, University of Bristol, Bristol, United Kingdom; 24. Centre for Metabolomics Research, Department of Biochemistry, Cell and Systems Biology, Institute of Systems, Molecular and Integrative Biology, University of Liverpool, BioSciences Building, Crown St., Liverpool, UK, L69 7ZB; 25. School of Epidemiology and Public Health, University of Ottawa, 600 Peter Morand Crescent, Ottawa, ON K2P 2C9, Canada; 26. Unit of Integrative Metabolomics, Institute of Environmental Medicine, Karolinska Institutet, Stockholm SE-171 77, Sweden; 27. Department of Respiratory Medicine and Allergy, Karolinska University Hospital, Stockholm SE-141-86, Sweden.

**Keywords:** Metabolomic Epidemiology, STROBE, STROBE-MetEpi, Reporting guidelines

## Abstract

Metabolomic epidemiology has expanded rapidly, but publications often lack sufficient detail for readers to assess study design, analytical methods, sources of bias, and the robustness and reproducibility of findings. Existing reporting recommendations in epidemiology and metabolomics do not fully address the specific challenges that arise when these fields are combined. To improve the completeness and transparency of reporting, we developed the Strengthening the Reporting of Metabolomic Epidemiology (STROBE-MetEpi) statement, an extension of the original STROBE guidance for observational research. The STROBE-MetEpi checklist includes 31 items and subitems covering the Title, Abstract, Introduction, Methods, Results, Discussion, and Other Information sections of metabolomic epidemiology studies. This explanation and elaboration document is intended to complement the STROBE-MetEpi statement by explaining the rationale for each checklist item and providing published examples of transparent reporting. It applies to studies using metabolomic profiling to explore human health, but not to randomized trials, methodological studies, reviews, or multi-omics studies. As with previous STROBE explanation and elaboration documents, its purpose is to improve how studies are reported, not to prescribe how they should be conducted. The STROBE-MetEpi statement and this accompanying document should support authors, reviewers, editors, and readers in improving the reporting, appraisal, interpretation, and reproducibility of metabolomic epidemiology research.

## Introduction

Metabolomic epidemiology, defined as “*the systematic use of epidemiological methods and principles to study population-based variation in the human metabolome as it associates with health-related outcomes or exposures”,* (Lasky-Su et al. 2021) has seen remarkable growth since emerging as a distinct field in the early 2000s (Lasky-Su et al. 2021; Fuller et al. 2023). The integration of mechanistic and biologically informative metabolomic data with epidemiological data holds great promise for increasing understanding of disease etiology and the discovery of novel biomarkers and therapeutic targets. However, moving toward translation has been hampered by a lack of transparency in how studies are conducted and analyzed, leading to difficulties in interpreting results and consequently, limited validation of pertinent findings (Zanetti et al. 2024; Considine and Salek 2019).

As metabolomic epidemiology represents the convergence of two distinct fields, it requires interdisciplinary design and analysis considerations that account for the multiple potential sources of bias and variation across both fields, as well as those that are unique to their combined use (Lasky-Su et al. 2021; Fuller et al. 2023; Zanetti et al. 2024). For example, metabolomic epidemiology must account for population factors that may confound the relationship between the metabolome and a phenotype, and how to modify standard multiple testing correction approaches to address the highly correlated nature of the metabolome. To fully understand how authors addressed these challenges, transparent reporting is key. Several groups have defined reporting guidelines for different aspects of the metabolomic epidemiology pipeline, such as the metabolomics standards initiative and the Metabolomics Quality Assurance and quality Control Consortium (mQACC) among others (Members et al. 2007; Considine and Salek 2019; Kirwan et al. 2018; Sumner et al. 2007; McDonald et al. 2022; Broadhurst et al. 2018; Kirwan et al. 2022; Goodacre 2007; Vinaixa et al. 2012; Du et al. 2022). However, these do not cover the full flow of a metabolomic epidemiology study, and evidence suggests that these are frequently not adhered to in metabolomic epidemiology manuscripts, with reporting remaining unclear and incomplete (Considine and Salek 2019; Mutter et al. 2019).

### Development, scope and intended use of STROBE-MetEpi

The original STROBE (Strengthening The Reporting of Observational Studies in Epidemiology) guidelines (Vandenbroucke et al. 2007; von Elm et al. 2007), which were published in 2007 and have been endorsed by >100 journals, aimed to improve the quality of reporting in epidemiological studies. We developed the STROBE Metabolic Epidemiology (STROBE-MetEpi) statement, an extension of STROBE providing a reporting checklist for metabolomic epidemiology studies from design to interpretation, as described in detail elsewhere (ref: pending dual publication). The scope of studies covered by these guidelines is shown in Box A. We consider these guidelines applicable to any study using the metabolome, lipidome, or exposome (when measured using Mass spectrometry or NMR spectroscopy) to explore, understand, and/or identify biomarkers of human health. This includes cohort, cross-sectional and case-control studies, studies within biobanks, and clinical metabolomics studies. We highlight that due to the additional complexities of integrating another omic with metabolomics, and the fact that the specifics of the integration will differ according to the additional omic type, we have not developed these guidelines to be applicable to multi-omic studies. However, we do note that the checklist items here may help to support the reporting of such studies or provide a reference for those seeking to develop a multi-omics reporting checklist.

Here we present a complementary elaboration and explanation document to accompany our checklist where we outline the need for the checklist, explain our rationale for the inclusion of each item and provide published examples of the items from the literature to facilitate understanding of the recommendations. These examples are selected based on the comprehensiveness and transparency of their reporting, rather than on the quality or relatability of the work itself, on which we make no judgement. Some examples have been edited for brevity by removing wording or citations not relevant to the example and to adhere to the current Journal style.

As with previous STROBE statements, this checklist is not intended as a prescription for how to conduct a study, but rather to ensure a comprehensive and transparent description of how that study was conducted. We advise authors to address all items somewhere in their paper to enhance the quality, completeness, and transparency of the reporting, but we do not advocate for a precise location or order. We have attempted to make the checklist comprehensive without being onerous for the authors. Further, we have tried to account for the differing technologies, biosamples, phenotypes and populations that can be leveraged for the field as well as the potential for ongoing technological and computational advances (Joshi et al. 2023; Fuller et al. 2023). By framing our guidelines as an extension to the STROBE statement, which has demonstrated success in improving the transparency of reporting across the field of epidemiology, we hope that uptake will be high. We will work with relevant journals to continually monitor its usage. For a glossary of terms used see **Supplementary Table S1.** The final checklist is shown in Table 1. The checklist items follow a generalized flow of a typical metabolomic epidemiology study (Figure 1)

### Literature Review

#### Selection of studies

To determine the need for our checklist, we examined current gaps in reporting practices in metabolomic epidemiology using a representative sample of the literature. Specifically, we reviewed how well existing metabolomic epidemiology literature adheres to items on our proposed checklist. Information specialists searched Embase, MEDLINE, and PubMed to identify 500 human metabolomic epidemiology papers published from 2019 onward. The searches focused on studies in which metabolomics was a main topic and prioritized the most relevant records for screening (see **Supplementary Methods** for full details of the search). These papers were narrowed down to the final 50 using the following inclusion criteria: metabolomic profiling by mass spectrometry (MS) or nuclear magnetic resonance (NMR) spectroscopy; analysis on 100 human biosamples. Multi-omic studies (metabolomics plus an additional omics), reviews/systematic reviews and meta-analyses of previously published studies were excluded.

#### Reviewer recruitment and scoring

Researchers in metabolomic epidemiology were then recruited through professional networks and social media, and 16 reviewers representing a range of career stages and areas of expertise were selected. Each reviewer was randomly assigned 11 manuscripts, ensuring that every paper was assessed independently by at least three reviewers. Using the checklist, reviewers rated reporting for each item as good, moderate, poor, or not applicable, depending on the relevance of the item to the study. Definitions and examples of poor, moderate and good reporting based on assessments from the primary STROBE MetEpi team were provided to the review team in advance to help them calibrate their ratings. Team meetings were held to discuss ratings and to refine the wording of the checklist.

#### Characteristics of included studies

The 50 included manuscripts represented a broad range of study settings, including populations from Europe, North America, Asia, and the Middle East, as well as substantial variation in sample size, biosample type, and analytical platform (Figure 2). Sample sizes ranged from 113 to 118,021 participants, and the number of metabolites or metabolite features analyzed ranged from 6 to 14,414. However, it was noted that some studies were ambiguous in their use of the term metabolite when they were actually referring to metabolite features. Seven studies did not report the total number of metabolites included in the analysis. Most studies used blood-derived samples, while others used urine, exhaled breath, follicular fluid, or muscle tissue.

Not all checklist items could be assessed in every paper; in such cases, the item was classified as not applicable. This occurred primarily when a specific analytical approach had not been conducted. Overall, 75% of checklist items were applicable across the 50 studies. Six items in the methods were considered nonapplicable by all reviewers for at least one paper. These related to signal intensity drift correction, model complexity choice, and pathway analysis. Eight items in the results section relating to subject follow up, multiple metabolite models, consensus determination when using multiple models, validation and pathway analysis, were also nonapplicable for at least one paper.

#### Main reporting gaps identified

The performance of each checklist item across the 50 papers is shown in Figure 3. The ratings varied by section and item. For the abstract, while papers generally established that they were using metabolomics, many did not provide information on analytical technology, biological specimen profiled nor size of the study. For the guidelines associated with the introduction and discussion sections of papers (guidelines 2–3 and 25–28), most papers were scored as ‘good’ by most reviewers. The percentage of papers scored as good was at least 84% for each item in the introduction; and from 69.7% to 94.4% in the discussion. The worst performance observed for the discussion was around the reporting of generalizability. With regards to the methods, the worst reporting was for sample handling and preanalytical processing, instrumentation and data acquisition, data processing, metabolite identification, and data QC and cleaning procedures (ranging from 22.2% to 62.9% as good). Reporting was better for the items pertaining to the statistical analysis of the data, apart from the handling of missing data (48.8% of studies rated as poor). In general, reporting of the results was better than for the methods, although many papers were rated as poorly reporting total number of identified metabolites and the number of metabolites which could be annotated (18.5% rated as poor). Reporting was also poor with regards to the number of subjects with missing information (30.9% rated as poor). Where pathway analyses were performed, reporting was rated as moderate or poor in more than half of cases. A majority of papers also performed poorly with regards to data and code sharing. The performance of each individual anonymized manuscript can be found in **Supplementary Figure 2**. It should be noted that the rating of checklist items varied across the reviewers. In some cases, a given checklist item was rated as “good” by one reviewer, but “moderate” or “poor” by another for the same paper. There were also instances where reviewers disagreed on whether a checklist item was relevant for a given paper. Disagreements were settled by an additional reviewer. This variability may reflect the differing expertise across reviewers, but it could also indicate inadequate reporting within the examined literature, which can make data extraction and interpretation challenging. With regards to the former, we did modify the original checklist with feedback from the reviewers to try to make it as accessible for non-experts as possible, however we note completion of the checklist will likely require input from co-authors covering a breath of experience.

When considered together, these results reveal that many crucial aspects required for contextualization and interpretation of metabolomic epidemiology literature are being under or inaccurately reported. The level of reporting detail differs across papers, and while this can be partly explained by limited word count, we also postulate this is largely due to a lack of standardization in how authors should be reporting. We propose that by following our checklist, we can begin to address this limitation and move the field forwards.

### Explanation and Elaboration of Checklist Items

Table 1 presents the STROBE-MetEpi checklist, organized according to the main sections of a scientific paper: Title and Abstract, Introduction, Methods, Results, Discussion, and Other Information. Some items include subitems, but not all subitems will apply to every study because their relevance depends on the study design, analytical platform, and objectives. The remainder of this Explanation and Elaboration document follows the checklist item by item and provides the rationale, reporting considerations, and illustrative examples for each item.

### 1. TITLE or ABSTRACT

The following information should be provided in the title and/or abstract

- Include the word ‘Metabolomic’, ‘Metabolite’, ‘Metabolome’ or similar
- State that the study is epidemiological or use epidemiological terms to make this explicit in the title or abstract. Specify the epidemiological study design.
- Provide information on analytical technology; biological specimen profiled and size of the study.

**Example:** Title

“Metabolomic profiles in breast cancer: a pilot case-control study in the breast cancer family registry.”

(Dougan et al. 2018)

**Example**: Abstract

**“Background:** Exposure to air pollutants including particulate matter (PM_2.5_), black carbon (BC), and carbon monoxide (CO), is linked to alterations in the human blood metabolome. We examined relationships between these exposures and the human milk metabolome, which may have implications for infant health. **Methods**: Human milk samples from 75 women participating in the Household Air Pollution Intervention Network trial in Jalapa, Guatemala were obtained during household visits at six months postpartum. Samples were analyzed for metabolomic profiling using liquid chromatography coupled with high-resolution mass spectrometry (LC-HRMS). 24-hour personal exposure to PM_2.5_, BC, and CO was measured during pregnancy at 9–19 weeks, 24–28 weeks, and 34–36 weeks gestation. Perturbations in the human milk metabolome associated with exposure to each pollutant during pregnancy were identified using an established untargeted metabolome-wide association study workflow. **Results:** We extracted 571, 492, and 690 metabolic features in the C18 and 93, 1381, and 909 features in the hydrophilic interaction liquid chromatography (HILIC) columns, for PM_2.5_, BC, and CO respectively (p < 0.05). 17 major metabolic pathways were observed to be associated with at least one of the pollutants, including pathways related to degradation of aromatic compounds, amino sugar and nucleotide sugar metabolism, and retinol metabolism. We annotated a total of 204 metabolites (level 1 confidence), of which 63 were associated with PM_2.5_, 84 were associated with BC, and 57 associated with CO. **Conclusions:** Household air pollution exposure from PM_2.5_, BC, and CO was associated with perturbations in metabolic pathways in human milk, including pathways related to inflammation and cellular signaling and repair.” (Hoyle-Gardner et al. 2026)

**Explanation:** Readers should be able to easily identify that metabolomic data play a central role in the study through the use of the term “metabolomic” or similar terms (“metabonomics”, “metabolome”). Although the term “epidemiology” (or “epidemiological”) does not need to be used, it should be made clear that the investigation is a metabolomic epidemiology study from the use of related terminology (e.g. cohort; case-control; cross-sectional). “Epidemiology” should at least be included as a keyword. Commonly used terms for the study design and for metabolomic data also ensure correct indexing of articles in electronic databases.

The abstract should provide an informative and balanced summary of what was done and what was found. This should be presented alongside critical issues in study design, definitions of exposures and outcomes, and the sources of metabolomic data. The abstract should include terms to help make the article discoverable as a metabolomic epidemiology study. Results should be presented in a fully transparent manner include quantitative metrics (e.g. point estimates and their error and p-value) wherever possible. The abstract should be sufficiently detailed to act as a stand-alone component of the manuscript. When sought or permitted by the journal, structured abstracts can provide clarity and help assure that all relevant information is included.

## INTRODUCTION

### 2. Background

Explain scientific background and rationale. Justify why metabolomic data are helpful to address the study question(s).

#### Example:

“Metabolomics, the systematic profiling of the small molecules in a biological system, represents a powerful tool to increase the understanding of the mechanisms of respiratory health, providing a downstream ‘snapshot’ of the status of a biological system reflecting phenotype as well as upstream genetic and environmental influences. As such, it is ideally suited to examine alterations in biological pathways that accompany phenotypical changes. Several studies have successfully used metabolomics to explore diseases including asthma and COPD, suggesting there are measurable and biologically informative alterations in the metabolome that reflect perturbations in the respiratory system. However, only a small number of studies have investigated the metabolome of forced expiratory volume in one second (FEV1) or the ratio of FEV1 to forced vital capacity (FEV1).”

(Kelly et al. 2022)

#### Example:

“Epidemiological studies of total physical activity (TPA) related to daily living and work have reported dose-dependent health benefits … However, the underlying mechanisms by which TPA exerts these various health benefits are poorly understood… Metabolic profiling using metabolomics in epidemiological studies allows us to comprehensively identify metabolic alterations in relation to lifestyle factors, such as physical activity, and to indicate potential biomarkers related to metabolic diseases… … However, although race differences in metabolic profiles have been reported…, no study has reported associations between metabolic profiling and daily physical activity in an Asian population, which are generally lean and have different dietary habits from Western populations”

(Fukai et al. 2016)

**Explanation:** The scientific background of the study provides important context for readers. It sets the stage for the study and describes its focus. It gives an overview of what is known on a topic and what gaps in current knowledge are addressed by the study. Background material should note recent pertinent studies and any systematic reviews covering the research area. Specifically, the introduction should justify the metabolomic approach to the research question and orient the reader as to what specific gap in the literature can be addressed by using metabolomic data.

### 3. Objectives

State the overarching objectives. Specify if this is a hypothesis-driven or discovery-based/exploratory study; if hypothesis-driven provide the predefined hypothesis. State whether this is a first report or a replication effort for existing results.

#### Example:

“we performed a systematic analysis of plasma metabolomics to 1) identify and validate metabolites associated with intake of total, caffeinated, and decaffeinated coffee and 2) prospectively evaluate the association of coffee-related metabolites with diabetes risk and the added predictivity of these metabolites for diabetes.”

(Hang et al. 2020)

#### Example:

“We hypothesized that the effect of blood lipid–related metabolites on primary open-angle glaucoma (POAG) would differ according to specific lipoprotein particles and lipid sub-fractions. The objective of this study was to investigate the associations between blood levels of lipoprotein particles and lipid sub-fractions and POAG”.

(Nusinovici et al. 2022)

**Explanation:** Setting the objectives at the very start of the manuscript orients the reader. It allows them to place the rest of the manuscript in context and to critically assess whether the objectives were achieved. It should also clarify whether the study is confirmatory, with a clearly defined *a priori* hypothesis, or exploratory. A significant proportion of metabolomic epidemiology studies are hypothesis-free or hypothesis-generating and will therefore often differ in their approaches and methods from those studies that aim to replicate an existing finding, such as a phenotype-associated metabolite or set of metabolites that predict a disease outcome. Therefore, it is vital to determine from the outset whether the study has a defined hypothesis. Replication and validation of findings represent a bottleneck in metabolomic epidemiology; highlighting whether the study was designed to address this issue will help to emphasize its importance.

## METHODS

### 4. Study design

Present the key elements of the study design and state whether the study was designed specifically for metabolomics.

#### Example:

“The Vitamin D Antenatal Asthma Reduction Trial (VDAART) was a randomized, double-blind, parallel-design trial conducted at three study sites across the United States …, to determine whether prenatal vitamin D supplementation lowers the risk of asthma in offspring. ….. Briefly, between October 2009 and July 2011, VDAART recruited pregnant non-smoking women aged 18–39 years who had a history of asthma, eczema, or allergic rhinitis, or whose partner (biological father of the child) had a history of these conditions. At 10–18 weeks gestation, 440 women were randomized to 4000 IU vitamin D daily, while 436 women were randomized to a daily placebo (both arms also received daily prenatal multivitamin containing 400 IU vitamin D). The outcome of interest was the composite measure of asthma or recurrent wheeze by age three years as described previously. …. The current analysis included women with plasma metabolomic data at two timepoints: study baseline (10–18 weeks gestation) and third trimester (32–38 weeks gestation), and their children, resulting in a final sample size of 685 mother-child pairs.”

(Huang et al. 2021)

#### Example:

“The Jackson Heart Study (JHS) was a single-site prospective population-based cohort study of cardiovascular disease and its risk factors among African American individuals. The study included 5306 African American participants aged 21 to 84 years residing in 3 counties surrounding Jackson, Mississippi. Participants were examined according to standardized protocols at baseline (2000–2004) and 2 follow-up visits. The JHS was approved by the institutional review boards of Jackson State University, Tougaloo College, and the University of Mississippi Medical Center. All participants provided written informed consent. …… The present study included 2346 participants from the JHS who received plasma metabolomic profiling after fasting for more than 8 hours and had no history of CHD.”

(Cruz et al. 2022)

**Explanation:** The authors should provide information on the overarching study design using epidemiological terminology, for example a cohort study or a case-control study. It should further specify the original purpose for which the samples were collected. If the study was designed to collect samples for metabolomics or were the samples originally collected for some other purpose as this may be relevant with regards to the suitability of collection and storage. To interpret subsequent results, it is useful to specify what aspects of the design specifically accommodate the needs of metabolomic analysis, and if any aspect of the design is non-optimal or challenging for metabolomic analysis. As appropriate to the study design, matching criteria and/or the use of follow up samples/longitudinal samples should also be explained. Report justification for sample size if relevant.

### 5. Study Setting and Participants

- Provide the eligibility and exclusion criteria for study entry, including setting, source population and sampling.
- Report on any participant or sample-specific characteristics that could affect the metabolome, if relevant.
- Provide details of ethics committee approval and participant informed consent, if relevant.

#### Example:

“We drew data from two large U.S. cohorts, the Nurses’ Health Study (NHS) and NHSII…..The NHS enrolled 121,700 female nurses aged 30–55 years in 1976, and the NHSII enrolled 116,429 female nurses aged 25–42 years in 1989…. Blood samples were collected from 32,826 women in NHS in 1989–1990 and 29,611 women in NHSII in 1996–1999. Women who provided blood samples had dietary and lifestyle profiles similar to those of women who did not. … First, we …drew participants with available plasma metabolomic data from previous sub-studies in the cohorts. After exclusion of participants who had a history of diabetes, CVD, or cancer prior to blood draw or had missing data on coffee consumption or metabolites, a total of 949 women with 427 named metabolites and 646 women with 413 metabolites were included in the discovery and validation sets, respectively. We developed a diabetes prediction model … Briefly, among participants with available metabolomic data, we identified incident cases of diabetes that occurred after blood draw until the end of follow-up (NHS, 30 June 2012, and NHSII, 30 June 2013). Using risk set sampling, we randomly selected three control subjects for each diabetes case subject among participants who were alive and free of diabetes at the time of diagnosis of the case subjects, matched on age at blood draw +−1 year, study cohort (NHS, NHSII), and fasting status…The study protocol was approved by the Institutional Review Board of the Brigham and Women’s Hospital and the Human Subjects Committee Review Board of the Harvard T.H. Chan School of Public Health.

(Hang et al. 2020)”

#### Example:

“The REGARDS study … enrolled 30,239 non-Hispanic Black and White participants ≥45 years of age between 2003 and 2007. … data on incident ischemic stroke cases…were compared to a stratified cohort random subset of participants. …. Briefly, participants were contacted by phone and consented individuals completed a telephone interview…. Exclusion criteria were medical conditions preventing long-term participation, past malignancies or active treatment for cancer, nursing home placement, inability to communicate in English, and race other than Black or White…. … During an in-home visit 2–3 weeks later, fasting baseline EDTA blood samples were collected by venipuncture…. For this analysis, we included all ischemic strokes adjudicated through April 1, 2019 (n = 1,075) without pre-baseline stroke or TIA and a cohort random sample that was stratified on age, race, and sex, (n = 968).….The REGARDS study was approved by the institutional review boards of all participating institutions and written informed consent was obtained from all participants. The metabolomics analysis was also approved by the Mass General Brigham institutional review board.”

(Ament et al. 2022)

**Explanation:** A detailed description of the study setting is vital for metabolomics studies since metabolite levels can vary by geography (due to differences in diet or genetics for example), season, and/or time of day, such as the diurnal pattern observed for cortisol levels (Edwards et al. 2001). Metabolites can also be influenced by setting-specific behaviors; hospitalized patients, for instance, smoke less while hospitalized and may therefore have lower levels of tobacco-related metabolites in their blood or urine. Metabolites are well-known to be affected by sample handling. This too can vary by setting, such as if hospitals process samples more quickly (or slowly) than samples collected in a home. All observational studies should describe setting in detail, but this is especially important for case-control studies where differences by case status could easily lead to biased results. In addition to the setting, the flow of participant selection and collection of participant-level data from biological samples and other sources such as questionnaires, clinical and health administrative records, physical measurements and wearable devices have implications for the analysis and interpretation of metabolomic studies and should be noted. As with any observational study, inclusion and exclusion criteria should be reported, and any differences in the operationalization between the compared groups documented. Many individual-level factors can also influence the metabolome, including but not limited to age, sex, pre- or post-menopausal status, body mass index, pregnancy (by trimester), diet, co-morbidities, use of prescribed and over the counter medication (inc. antibiotics), so all such pertinent factors should also be reported upon, and directed acyclic graphs may be of use to demonstrate how such factors are relevant to the research question of interest. Where references are used to provide additional information on the study setting and participants, e.g. detailed information on cohort recruitment and retention, authors should ensure these references take the reader to the primary source. As with all human studies details of ethical approval and informed consent should be provided if relevant in accordance with the Helsinki Declaration.

### 6. Biological Sample Collection, Handling and Storage

- Report on the biological sample type and collection protocol including timing and conditions in sufficient detail to assess quality of sample. Specify whether conditions were the same for every sample in the study.
- Report on sample storage prior to processing.

#### Example:

“Participants had blood drawn at a community medical laboratory using standard blood collection techniques. Participants were not required to fast. Samples were stored at 4 °C before shipment to the processing laboratory the following day. Samples were processed according to BCGP’s Standard Operating Procedure. EDTA vacutainer tubes were centrifuged for 10 min at 1300g/4 °C/with brake on to separate out the plasma fraction. Plasma was transferred to a cryovial and stored at − 80 °C between 19 to 24 h after laboratory blood collection. All samples were noted to be free of hemolysis and lipidemia”.

(Qi et al. 2021)

#### Example:

“For the collection of saliva samples, a hygienic extraction system (Salivette^^®^^; Sarstedt, Nürnbrecht, Germany) was used. The subjects were instructed to chew on a plain cotton roll to stimulate salivation. After exactly 1 min, the rolls containing the absorbed saliva were placed into the Salivette^®^ and centrifuged immediately at 1000 g for 20 min at 4°C. The ejection of food remnants, cell debris, and insoluble materials led to a supernatant, which was stored at −80°C until further analysis”.

(Andorfer et al. 2021)

#### Example:

“For each subject, first morning fasting urine was collected in sterile containers and transported to the laboratory (only those samples with a collection time shorter than 3 h were considered). The urinalysis included general urine test, albuminuria, and creatinine. For urine NMR metabolomics analysis, a centrifugation step was performed to remove cell debris (13 000 × g, 5 min, and room temperature). Then, the supernatant was aliquoted and stored at − 80°C until analysis (maximum storage time of 9 months)”.

(Lucio-Gutierrez et al. 2022)

**Explanation:** Sample collection procedures can influence the resulting metabolomic profiles (Smith et al. 2020). Providing information on the collection of samples and the way in which they were stored prior to processing allows the reader to assess the quality of the samples and to identify any potential sources of bias. Therefore, it is of particular importance to report whether all samples were collected in the same way, or if there could be any systematic bias. Ideally, information should include the Standard Operating Procedure covering; fasting status, where the samples were collected, time of day and season, who did the collection, what exactly was collected (e.g. for urine; spot urine or 24 h collection), what volume was collected, what were the handling conditions, what type of tube were the samples collected in, was anything added (e.g anticoagulants), how were the samples shipped to the lab from the collection center, and what were the storage conditions in the lab, including temperature and time until freezing. A common problem in the collection of blood is hemolysis, that is the breakdown or destruction of red blood cells so that the contained oxygen-carrying pigment hemoglobin is freed into the surrounding medium (Yin, Lehmann, and Xu 2015). If this occurred it should be reported on, and those samples excluded or, if possible, corrected for. It is also important to provide the lengths of the intervals between dates of recruitment, sample collection and processing. In summary, the main aim of this section is to provide sufficient and necessary detail to establish whether the samples collected were fit for the purpose of metabolomics.

### 7. Pre analytical Biological Sample Processing and Preparation

Provide sufficient detail to enable replication of the pre-analytical processing conditions prior to metabolomic profiling. If referencing an existing Standard Operating Procedure (SOP), provide DOI, version control and any deviations.

#### Example:

LC-MS sample preparation; “All serum samples were thawed on ice at 4 °C followed by deproteinization by the addition of methanol (1:3 ratio of serum to methanol, room temperature), vortex mixed for 15 s and centrifuged for 15 min at 15871 xg. The supernatants were transferred to new Eppendorf tubes, lyophilized at 45 °C for 16 h (HETO VR MAXI vacuum centrifuge attached to a Thermo Svart RVT 4104 refrigerated vapor trap; Thermo Life Sciences, Basingstoke, U.K.) and stored at 4 °C prior to analysis. The samples were reconstituted in HPLC grade water (1:1 ratio of original serum volume to water), vortex mixed and centrifuged for 15 min at 15871 xg. The supernatants were transferred to analytical vials, stored in the autosampler at 4 °C and analyzed within 48 h of reconstitution”

(Zelena et al. 2009)

#### Example:

GC-MS sample preparation; “Mitochondrial pellets corresponding to 1 mg protein and half of the volume of each corresponding cytoplasm were applied to metabolic profiling in technical triplicate. The results were reproduced by analyzing different biological preparations. The cytoplasm was dried in a Concentrator plus vacuum rotator (Eppendorf, Germany) before 1 mL extraction medium (− 20 °C methanol/water (v/v: 90/10) containing 1 μg/mL of β-phenylglucose and ribitol as internal standards) was added to each mitochondrial or cytoplasmic pellet. Samples were incubated at 4 °C and 1200 rpm for 10 min after short vortex. After centrifugation at 21,000 ×g and 4 °C for 10 min, the metabolite-containing supernatants were transferred to new reaction tubes and dried in a Concentrator plus vacuum rotator and stored under nitrogen atmosphere at − 80 °C until derivatization.. Based on the protocol of Fiehn (2006), sample derivatization GC-MS analysis and data evaluation were performed as described by Pan et al. (2016). Briefly, the prewarmed pellets were derivatized by shaking with 20 μL 20 mg/mL methoxyamine hydrochloride (Sigma-Aldrich, Germany) in pyridine (Sigma-Aldrich, Germany) at 1200 rpm and 28 °C for 90 min and, after short centrifugation, with 50 μL N-methyl-N-trimethylsilyltrifluoroacetamide (Sigma-Aldrich, Germany) at 1200 rpm and 37 °C for 30 min”.

(Pan et al. 2018)

#### Example:

NMR sample preparation; “Protein was removed from 200 μL of each serum sample using a BioVision Deproteinizing Sample Preparation Kit (Milpitas, CA, USA). The kit utilizes perchloric acid to precipitate protein from the sample; precipitated protein was removed by centrifugation. A neutralizing solution was added to supernatants to neutralize pH. Prior to NMR analysis, sample pH was adjusted if necessary, using hydrochloric acid and sodium hydroxide. During the extraction process, there was a loss of volume; samples were brought to 190 μL with water, and then 10 μL of 5 mM 2,2-dimethyl-2-sila-3,3,4,4,5,5-hexadeutero-pentane sulfonic acid (DSS-d_6_, Chenomx Inc., Edmonton, Alberta, Canada) was added as a concentration reference and chemical shift indicator. Samples were centrifuged to remove any residual particulate matter. The clarified, protein-free serum samples were then transferred to 3 mm NMR tubes”.

(Reinke et al. 2013)

**Explanation:** Pre-analytical variability can introduce bias into the subsequent analyses particularly when sample handling varies systematically by case status (Hernandes, Barbas, and Dudzik 2017). Consequently, it is important that information pertaining to the handling and preprocessing steps is accurately reported. The exact items to report are dependent on sample type and planned analytical method (details in 8. Instrumentation and data acquisition). Detailed pre-analytical reporting standards have been previously published (Sumner et al. 2007). We recommend that high-level information should be included in the main text, examples of such pertinent information is shown in Box B.

### 8. Instrumentation and data acquisition

Provide sufficient detail to enable replication of the analytical method used to collect the metabolomics data, including name and version of instrument, as well as the software version used to control the instrument. If referencing an existing SOP, provide DOI, version control and any deviations.

#### Example:

LC-MS instrumentation; “All analyses were performed with a UPLC system (UPLC Acquity, Waters Ltd. Elstree, U.K.) coupled online to a TOF mass spectrometer (LCT Premier, Waters MS Technologies, Ltd., Manchester, U.K.). For high sensitivity, the instrument was operated in V mode, with DRE and lock mass correction. Optimized UPLC and MS settings for analysis in the ESI+ and ESI− modes are shown in the Supporting Information”. (Zelena et al. 2009) (See Box C for details)

#### Example:

GC-MS instrumentation; “Derivatized samples were analyzed using Agilent 7890A GC coupled to a Leco Pegasus HT TOF mass spectrometer applying the same chromatographic and MS parameters previously described”

(Dahabiyeh et al. 2024)

The paper above referenced the existing protocol published in Barupal et al (2019): An Agilent 6890 gas chromatography instrument equipped with a Gerstel automatic linear exchange systems (ALEX) which included a multipurpose sample dual rail and a Gerstel cold injection system (CIS). The CIS temperature program was: 50 °C to 275 °C final temperature at a rate of 12 °C/s and held for 3 min. Injection volume was 0.5 μL with 10 μL/s injection speed. Injection mode was splitless with a purge time of 25 seconds. Injector liner was changed after every 10 samples. Injection syringe was washed with 10 μL of ethyl acetate before and after each run. A Rtx-5Sil MS column (30 m length, 0.25 mm i.d., 0.25 microM 95% dimethyl 5% diphenyl polysiloxane film). An additional 10 m integrated guard column was used. Mobile phase was 99.9999% pure Helium gas with a flow rate of 1 mL/min. GC temperature program was: held at 50 °C for 1 min, ramped at 20 °C/min to 330 °C and then held for 5 min. A Leco Pegasus IV time of flight mass spectrometer was used to acquire data. The transfer line temperature between gas chromatograph and mass spectrometer was set to 280 °C. Electron ionization at −70 V was employed with an ion-source temperature of 250 °C. Acquisition rate was 17 spectra/second with a scan mass range of 85–500 Dalton (Da).”(Barupal et al. 2019)

#### Example:

NMR instrumentation; “One-dimensional ^1^H NMR spectra were acquired using a 600 MHz Varian Inova spectrometer using a Varian Unibody 3 mm 1H19F probe (Varian Inc., Palo Alto, CA, USA). A tnnoesy pulse sequence (circa Vnmr 6.1B software, Varian Inc.) with the following parameters was used to acquire all spectra: preacquisition delay of 1 s, mixing time of 0.1 s, acquisition time of 4 s, sweep width of 7200 Hz, 256 transients, 30 °C. Metabolite identification and quantification of 1D spectra was achieved using the 600 MHz database provided in Chenomx NMR Suite Professional software v5.1 (Chenomx Inc., Edmonton, Alberta, Canada). CAS Registry numbers and chemical shifts are presented in Supplementary Table S1”

(Reinke et al. 2013)

**Explanation:** Provide enough detail that anyone with a working knowledge of the type of instrument (e.g., MS-based or NMR) could replicate the analytical method used to collect the metabolomics data. This should include the name of the instrument along with the version, as well as the software version used to control the instrument.

These details should be reported in the main text, supplementary information or with reference to an existing protocol/Standard operating procedure (SOP). The SOP should have a DOI or version control and if referencing an existing protocol any deviations from this protocol must be detailed. Note most papers will usually report these details briefly in the main text and supplementary information is expected to contain the full operating process. The exact information included will depend partly on the technology used. We refer readers to Sumner et al (2007) (Sumner et al. 2007) for detailed guidance on reporting instrumentation and data acquisition.

### 9. Data processing

Provide sufficient detail to enable replication of the transformation of raw data into a data table suitable for analysis, including software package and version number and parameter settings used

#### Example:

“All raw data files were converted to NetCDF format using the Waters DataBridge software on a Windows PC. The freely available XCMS software was employed to convert (or deconvolve) each 3-D data matrix (intensity Å~ m/z Å~ time - one per sample) into a matrix of detected peaks vs sample identification (ID)… Default settings were employed in XCMS with the exception of S/N threshold [3], mass limit [0.1 amu], time limit [15 s], and sample limit [3]” (Zelena et al. 2009) (see Box C)

#### Example:

“NMR sample preparation and spectra acquisition…H NMR spectra were processed using the TopSpin 3.6.1 (Bruker Biospin, USA) software with LB = 0.1 Hz, EM, manually phased, baseline-corrected (ABSN function), and referenced to the TSP singlet at δ 0.0 ppm. The identification of metabolites in the NMR spectra was performed with Chenomx NMR Suite 8.5, in combination with data extracted from HMDB (https://hmdb.ca), and BMRB (https://bmrb.io/) databases.… qNMR urinalysis…The ERETIC2 module, available in TopSpin software, was used to proceed with metabolites quantitation through both the 1H NMR spectrum (qHNMR) and sucrose calibrant… Chemometrics data pre-processing… The 1H NMR data from urine samples of patients and controls, a total of 60 spectra, were exported from TopSpin software in JCAMP format, and imported to MATLAB R2020b (The MathWorks, Inc., MA, USA)… Changes in peak positions of the same analyte signal between samples were corrected using the icoshift algorithm.. Subsequently, data were corrected of unwanted variations applying the following methods available in the PLS-Toolbox software”

(Lucio-Gutierrez et al. 2022)

**Explanation:** There are many software packages available for processing raw spectral files and converting them into a data matrix of metabolite abundances for each sample. Each software package (and version) has different processing parameters that can be set and, in some cases, raw instrument files must be converted to an open-source format prior to spectral processing. Providing details on all software packages and parameter setting used enables the reader to assess potential sources of bias arising from this process. If referencing an existing protocol, any deviations from the protocol should be detailed. Reference to default settings without providing details should be avoided, as these settings may not be readily accessible to the public, especially if proprietary software is used. Screenshots of settings may be helpful here.

Deconvolution is an important step to transform NMR and MS data into lists of metabolites/metabolite features and abundances of these feature and information on how data were cleaned prior to analysis should be reported. For NMR this includes: bin size with rationale (if appropriate); deconvolution using instrument or third-party software. In this case enough information should be provided to allow someone to replicate the deconvolution step. For [G/L]C-MS the information on deconvolution using instrument or third-party software is required along with the parameters used during deconvolution. For absolute quantification of key metabolites provide details of how this was achieved, this can include external calibration or internal calibration with (e.g.) isotope standards or employment of the standard addition method (SAM). Such information is critical because absolute concentration values should not be extrapolated or interpreted without understanding the underlying calibration method and its assumptions. Proper reporting ensures that the results are accurate, reproducible, and comparable across studies.

We note that while these steps are discrete, they are often performed in a single pipeline (e.g. compound discover). However, they do not necessarily need to be reported in the order denoted here.

When metabolomic data are generated at multiple labs or time points or data are to be combined across different studies, pooled samples or bridging samples will commonly be used to facilitate comparison across studies. The samples, including the analysis of reference standards (e.g., National Institute of Standards and Technology Standard Reference Materials) and any statistical techniques used to make data comparable should be described.

### 10. Metabolite identification/annotation

Describe how metabolite identification or annotation were achieved, including which physicochemical properties the decisions were based on and which reference libraries were used.

#### Example:

“Peak deconvolution and metabolite identification were performed using Agilent TOF-Quant software (version B.07.00, Agilent Technologies) as described. To ensure accurate metabolite identification, metabolites were matched against retention time, accurate mass, and MS/MS fragmentation patterns of 408 chemical reference standards in an in-house database. Metabolites were only included for statistical analysis if the accurate mass, retention time, and MS/MS fragmentation pattern matched to an authentic standard; thus, all metabolites reported have a Level 1 identification level as defined by the Metabolite Standards Initiative”

(Reinke et al. 2022).

#### Example:

“Metabolite identification/annotation was performed by matching signals to both an in-house RT, exact mass, MS/MS reference library and public mass spectral databases. The in-house library contained over 2400 metabolite standards at that time. Peaks were matched with accurate mass (±5 ppm), retention time (±0.5 min), and MS/MS spectral similarity (>30%). For public databases, peaks were matched to NIST, ToxCast, and HMDB databases, and matching was based on accurate mass (±5 ppm) and MS/MS spectral similarity (>30%). A previously described ontology system was used to label each match according to the evidence supporting the metabolite assignment. OL1 represented the highest ontology level, indicating a match by exact mass (MS), MS/MS fragmentation pattern, and retention time (RT) to the in-house library. Features assigned as OL2a matched by exact MS and RT, while OL2b matched by MS and MS/MS spectra. For public database matches, PDa indicated a match by MS and experimental MS/MS spectra from NIST. PDb represented a match based on MS and theoretical MS/MS spectra from HMDB, while PDc included matches based on MS and isotopic similarity. Lastly, PDd indicated a match by MS alone. Due to the limitations of untargeted metabolomics, structural isomers, particularly those differing in D- and L-configurations, are not always distinguishable within the analytical platform used in this study. Matching of peaks to the in-house libraries and public databases, and automated assignment of ontology labels, was performed using ADAP-KDB”

(Lynch et al. 2025)

**Explanation:** A recent study reported that a majority of papers do not provide sufficient information on how identification/annotation was performed, posing an “existential threat” to discovery metabolomics (Theodoridis et al. 2026). To establish the level of certainty associated with the reported metabolite identification/annotation, sufficient detail supporting the level of certainty needs to be provided, ideally with reference to international standards (e.g., the Metabolomics Standards Initiative (MSI)) (Sumner et al. 2007; Schymanski et al. 2014). For mass spectrometry, identifications/annotations are typically based on comparing and matching indicators of physicochemical properties of the observed peak (such as accurate mass, retention time, and fragmentation patterns) to those of known compounds in chemical libraries (either available databases or in-house libraries) when available. Accordingly, it is important to report sufficient detail to convey the certainty of the reported identification/annotation. This should include the basis of the identification/annotation (in-house chemical standard versus external source). The observed indicators of metabolite physicochemical properties of the metabolite should be reported in relation to the source of the identification/annotation. Any additional details including isotopic pattern, adducts, and biological plausibility can also be included to support identification/annotation. For the list of reported metabolites, a standardized unique chemical identifier should also be included to avoid confusion arising from the use of common or alternate metabolite names (e.g., InChIKey, HMDB ID, PubChem ID, ChEBI ID). See an example in Box D. Chemical structural information should not be indicated when unknown (e.g., stereoisomers, regioisomers). For NMR-based annotations, chemical shifts and peak cluster patterns should be reported, as well as providing sufficient supplementary information on the 2D experiments performed, when novel annotations are achieved. When appropriate, confirmation using spiking experiments with authentic chemical standards should also be included to verify the spectral features.

We note that identification and annotation can be a dynamic process as new information becomes available on unknowns via subsequent analyses. In addition, reporting guidelines such as the MSI are continually being updated. For further discussion of the challenges of metabolomic identification and annotation, we refer to readers to (Theodoridis et al. 2026)

### 11. Quality assurance, signal intensity drift correction, quality control, and data cleaning procedures

- Describe all quality assurance, signal intensity drift correction, quality control, and data cleaning procedures; how blanks, system suitability mixes, and quality control samples were prepared and applied before and during data acquisition.
- If signal intensity drift correction was performed, reference should be made to the algorithm used.
- For quality assessment and data cleaning after data acquisition, include which metrics and/or exclusion criteria were used and how they were applied.

#### Example:

“Several types of controls were analyzed in concert with the experimental samples: a pool of well-characterized human plasma served as a technical replicate throughout the dataset; extracted water samples served as process blanks; and a cocktail of quality control standards that were carefully chosen not to interfere with the measurement of endogenous compounds were spiked into every analyzed sample, allowed instrument performance monitoring and aided chromatographic alignment. Instrument variability was determined by calculating the median relative s.d. for the standards that were added to each sample before injection into the mass spectrometers. Overall process variability as determined by calculating the median relative s.d. for all endogenous metabolites (that is, noninstrument standards) present in 100% of the pooled matrix samples was 10%. Experimental samples were randomized across the platform run with quality control samples spaced evenly among the injections.”

(Pietzner et al. 2021)

#### Example:

“A QC based on pooling together equal aliquots from each of the 84 samples was also prepared according to both methods. … All features with a relative standard deviation (RSD; standard deviation of the peak area divided by the mean peak area across QC samples) were excluded to maximize feature reproducibility, resulting in the retention of 81% (15,158/18,822) of HILIC mode features and 94% (12,023/12,794) of RPLC mode features.”

(Rosen Vollmar et al. 2023)

#### Example:

“A single pooled quality control (QC) sample (intra-study QC sample) was prepared by pooling 200 μL aliquots of plasma from each sample followed by vortex mixing for 60 s. Twenty-two (DS) and nineteen (VS) 200 μL aliquots of the intra-study QC sample were transferred to separate Eppendorf tubes for extraction as described above for the plasma samples. … Signal correction was performed to minimize run-order associated drift in response by applying quality control-based robust LOESS (locally estimated scatterplot smoothing) signal correction (QC-RLSC) (Dunn et al., 2011a, b). Quality assessment and quality-based filtering of the data were performed applying the data acquired for QC samples only. The relative standard deviation (RSD) for QC samples only (from QC sample injection nine onwards) and the percentage of QC samples where a response was reported were calculated for each metabolite feature. Data for all biological samples and QC samples for all metabolites with a relative standard deviation for response > 20% in the studied QC samples and after QC-RLSC were removed from the dataset prior to statistical analysis. Data for all biological samples and QC samples for all metabolites which were detected in < 60% of the studied QC samples were removed from the dataset prior to statistical analysis.”

(Chacko et al. 2021)

**Explanation:** Providing information on quality assurance and quality control (QC) procedures enables the reader to assess potential sources of bias arising from data acquisition. Ideally, details on any procedures taken to reduce bias or random error during data acquisition should be clearly described. These include whether sequence randomization was performed to avoid confounding of intra- or inter-batch drift with exposures or outcomes or for performing batch median normalization. Details on how pre-acquisition instrument quality assurance checks were performed should also be provided. QC samples are commonly applied in metabolomics to monitor and correct for instrument drift, and to assess resulting data quality (Broadhurst et al. 2018). QC samples can be prepared in various ways, depending on the biological matrix used and sample amount available. Authors should explicitly report on how QC samples were prepared and how frequently they were included in the data acquisition sequence. If signal intensity drift correction was performed to remove intra- and inter-batch bias, details on the algorithm used should be included. For quality assessment and data cleaning, include which metrics were used and how they were applied. If available, authors are encouraged to report resulting quality assessment metrics for each relevant metabolite. Note that some commercial labs may not provide comprehensive quality assurance and quality control information, which hinder sufficient reporting. We refer to readers to the Metabolomics Quality Assurance and Quality Control Consortium (mQACC) for an in-depth discussion of QA in untargeted metabolomics. (Mosley et al. 2024)

### 12. Transformation/Scaling

If transformation or scaling were applied prior to statistical analysis, describe which methods were chosen.

#### Example:

“To reduce bias due to outliers and skewed distribution, inverse normal transformation was performed on each metabolite for subsequent analysis”

(Cao et al. 2023).

#### Example:

“The metabolic measure was standardized as implemented in the Numero function nroPreprocess(method = ”standard”), which log transforms skewed data, truncates extreme outliers and scales by standard deviation”.

(Makinen et al. 2023)

#### Example:

“Data were log-transformed, both to stabilize variance and to approximate the multivariate normal distribution needed for parametric univariate and multivariate statistical modeling … Prior to principal component analysis (PCA) and hierarchical cluster analysis (HCA), metabolite profiles were scaled to unit variance (auto-scaled), which allows each metabolite to be compared within the analysis with no bias because of differences in absolute concentration variance”

(Makinen et al. 2023).

**Explanation:** Metabolomic data tend to be highly skewed and often sparse, violating the normality assumptions of commonly used statistical analysis approaches. Similarly, as much as a 5000-fold difference in measured levels can exist across the metabolites in a dataset, but these differences are not proportional to their biological relevance, as metabolites present in high concentrations are not necessarily more important than those present at low concentrations (van den Berg et al. 2006). Data pretreatment methods aim to deal with these issues to aid in the analyses and interpretation of metabolomic data. There are three main types of pretreatment methods: transformation to deal with non-normality of data or heteroscedasticity; centering, which converts all the levels to fluctuations around zero to offset differences between high and low abundant metabolites; and scaling, which divides each metabolite level by a scaling factor to make differences in levels relative to a standardized metric (e.g. divided by standard deviation) (van den Berg et al. 2006). There are a multiple options available for each type, either applied directly by the researchers or using a publicly available pipeline. No gold standards have been agreed upon, largely because the optimal approach depends upon the structure and properties of the metabolomics dataset and the biological questions to be answered, as well as the statistical methods used to answer these questions (van den Berg et al. 2006; Li et al. 2016). Therefore, reporting these parameters provides information on the dataset and is vital to understand the validity of the overall statistical approach and interpretation of its findings.

### 13. Handling of missing data

Describe how missing values were treated for both the metabolomic and covariate data. If imputation was performed, describe the chosen method.

#### Example:

“Missing values were imputed using the R package MICE with default settings (five imputations applying predictive mean matching)”

(Sekula et al. 2016)

#### Example:

“Metabolites with coefficient of variation (CV%) > 25% or missing > 75% were then excluded. Remaining missing values were imputed using the k-nearest-neighbor imputation method (R package “VIM”).”

(Mendez et al. 2024)

**Explanation:** Missing data are extremely common in mass spectrometry based metabolomic profiling, with missingness of >80% for some features (Wei et al. 2018), complicating analyses and interpretations (Kokla et al. 2019). In metabolomics, missingness may result from true absence within a given sample, presence at a level below the Limit of Detection (LOD) or Quantification (LOQ) or from technical issues arising from inappropriate transformation of the measured signal to the numerical data format (Kokla et al. 2019; Shahjaman et al. 2021). Currently, there is no gold standard approach for the imputation (that is the replacement of missing values with information available from the existing data (Kokla et al. 2019). In some cases, samples or metabolites may be excluded based on missingness, particularly in the case of exogenous metabolites such as food or drug metabolites that may only be present in a small proportion of the populations (Davis et al. 2022). The optimal choice depends on the type of missingness, the proportion of missingness, the preceding data preprocessing steps, the presence of any outliers, as well as the assumptions of the statistical method used to analyze the data (Wei et al. 2018; Kokla et al. 2019; Yuan et al. 2024).

The handling of missing data can have important implications for downstream statistical analyses and results inferences (Kokla et al. 2019). Being transparent about how metabolites were treated, and whether all metabolites were treated the same way, is vital for the reader to interpret the results. For example, how to interpret a highly significant metabolite finding if that metabolite was imputed in a large percentage of the population. It is also necessary to report how missing covariate data were treated, again for the purpose of interpretation of findings. If there were no missing data for either the metabolomic or covariate data, this should also be stated.

### 14. Type of model

Provide a clear summary of the model and the framework in which it was performed, specifying dependent and independent variables and covariates as appropriate.

#### Example:

“A Cox regression analysis was performed to examine the association between metabolite levels (independent variable) and incident CHD (dependent variable), and linear regression analysis was used to assess the association between metabolite levels (independent variable) and log (CAC score + 1) (dependent variable). Metabolite levels were standardized to multiples of 1 SD. Two sets of covariates were used in the incident CHD and CAC analyses. The first set included age, sex, and batch as covariates; the second set included age, sex, batch, and traditional clinical risk factors as covariates.

….All statistical analyses were performed using R software, version 3.3.1.

To assess the significance of differences in β coefficient estimates between the JHS and WHI cohorts, a zscore was created for each metabolite comparison by dividing the difference in β estimates in the 2 cohorts by the square root of the sum of the variances. Because the β estimates were normally distributed in each cohort, the P value for this difference was calculated using the probnorm function in SAS software, version 9.4 (SAS Institute Inc), with the significance level set at P < .01 owing to the exploratory nature of the analysis.”

(Cruz et al. 2022)

#### Example:

“We first carried out analyses in the MESA study data and then took forward significantly associated features for replication in the Rotterdam Study and LOLIPOP data. In MESA, for each of the 30 590 spectral features, we carried out a linear regression against each of CAC [coronary artery calcium] and IMT [carotid intima-media thickness] with adjustment for age, gender, ethnicity, and measurement phase (Model 1). Discovery models used partial Spearman correlations between CAC or IMT and metabolic features along with P values to indicate the strength of associations. We also presented results as standardized regression coefficients (per standard deviation) from linear regression…”

(Tzoulaki et al. 2019)

#### Example:

We aimed to estimate the direct effects of (dh)ceramides on cardiometabolic disease risk that could not be attributed to the influence of related ceramide metabolites. Therefore, we developed a graphical model-based method, the NetCoupler-algorithm. In a first step, we estimated a network model of conditional dependencies, where edges represent covariance between two (dh)ceramides that could not be explained by adjustment for any subset of other (dh)ceramides. To this end, we applied an order-independent implementation of the causal structure learning PC-algorithm. The resulting network graphically encoded the family of causal models that could have generated the observed conditional independence structure, i.e., the skeleton of the data-generating DAG. This conditional independence network was then used to detect links between individual metabolites and disease incidence that could not be explained by confounding influences through other (dh)ceramides. By definition, at least one subset of direct neighbors is sufficient to block confounding from the whole network. However, sufficient adjustment sets could not be unambiguously read from the graph because the edges were not directed. Therefore, the NetCoupler-algorithm iterates for each metabolite through adjustment for all possible combinations of direct network neighbors. A metabolite is then only classified as a direct effector if the association with disease incidence is robust across all these sub-models.”

(Wittenbecher et al. 2022)

**Explanation:** Studies should provide a clear summary of the model(s) to be performed and the framework in which it is to be performed. This may include traditional statistical models, prediction-based approaches, and network analyses. The specific type of statistical model (linear models, mixed models), along with distributional assumptions and model parameters should be provided (e.g. Poisson, normal, binary). Both the dependent and independent variables as well as covariates/confounders should be stated. Additional analyses that further explore the findings (e.g. stratifications, interactions, secondary outcomes) and plots/graphs that are relevant should be described (e.g. Kaplan Meier Curve). Any other statistical approaches to combine and/or summarize the findings should also be detailed.

### 15. Model complexity choice/hyperparameter optimization/model validation

- If relevant, describe the assumptions that pertain to your chosen model and how these assumptions were tested.
- Describe how the model was optimized including (as appropriate) formal model selection procedures, model tuning, optimizing number of latent variables, covariate selection; and state why these choices were made.
- State the software used, its version, and the procedure applied.

#### Example:

“Evaluation of the predictive powers and tuning parameters for each prediction model was based on fivefold cross-validation. In fivefold cross-validation, all the subjects were randomly split into five roughly equal-sized groups. We predicted each group (test dataset) using the model fitted on the remaining four groups of data as training datasets. We repeated this process for each of the five groups and obtained five estimates of predictive power. This cross-validation for evaluating predictive power was referred to as the “outer loop”. Predictive power was evaluated based on the predictive correlation coefficient (i.e., Pearson’s correlation coefficient between the predicted and measured CES-D scores) for quantitative traits and the area under the curve (AUC) for binary traits. The parameters for the prediction models (i.e., hyperparameters) were also selected based on fivefold cross-validation. Training datasets from the outer loop were split into five parts again, and each group was used as a test dataset; the remaining four parts were used as training datasets repeatedly. The mean of the estimates of the five predictive powers were calculated, and the parameters that gave the maximum predictive power were used as the optimized parameters. This cross-validation is also known as the “first inner loop”. If the prediction models consisted of separate algorithms for feature selection and prediction, the parameters for the feature selection were tuned in the first inner loop; then, the training datasets from the first inner loop were further split into five datasets, and fivefold cross-validation was performed yet again to select the optimized parameters for the prediction part (the “second inner loop”). The subject sets included in the outer loop, the first inner loop, and the second inner loop were common in all the prediction models”

(Takahashi et al. 2020).

#### Example:

“Prediction Models… we constructed receiver operating characteristic (ROC) curves and calculated C statistics … in the JHS cohort, and we aimed to replicate these findings in the WHI cohort… We corrected for optimism in the JHS cohort to account for overfitting due to testing in the derivation cohort by using the validation function of the rms package in R software, version 6.2–0 (R Foundation for Statistical Computing)…. We constructed a metabolite risk score…[and used a] Cox model regularized by Ridge penalties … to predict incident CHD–free survival”

(Cruz et al. 2022)

#### Example:

“In the unmatched random forest analysis comparing individuals with a prospective 1–5 years MDD diagnosis against controls, data were first split into a training set (90% of individuals) and independent final test set (the remaining 10% of individuals). The training set was subjected to a 10-fold cross-validation procedure to determine whether a model of predictive value could be produced. During this cross-validation, a feature selection step was applied on the training data. Specifically, an initial, “dummy” random forest was produced, from which the top 5% of predictor variables were shortlisted, and used to generate a second random forest model which was used to test the independent final test set. As the number of control individuals outnumbered those with a prospective MDD diagnosis, a random subset of the control group was extracted prior to each 10-fold cross-validation. This was repeated 10 times, resulting in an ensemble of 100 models from which the mean ROC AUC and most important predictor variables were determined. This process of creating multi-layered random forests is believed to be particularly adept at exploring a larger parameter space quickly in high dimensional data. As a secondary measure to ensure models were not overfitted, a model with randomly permuted classes was fitted and tested in parallel. A mean ROC AUC of ~0.50 was indicative of a null distribution, to which the predictive models were compared. Finally, the entire training set (90%) was used to predict the independent test set (10%) where class sizes were not matched.”

(Radford-Smith et al. 2023)

**Explanation:** All machine learning / AI / chemometrics models are at risk of overfitting and include parameters which need to be tuned. A valid model is usually considered to be one for which its power to fit/predict new data is similar to the data on which it was trained. Typically, parameters are tuned using cross validation to check predicting success, and further cross validation may be done to estimate final predictive ability. Many different train/test splitting procedures are available, and it is important to report clearly what approach was taken. Often models developed on a discovery cohort will be checked in a validation cohort, though this does not obviate the need to report parameter tuning in the discovery cohort.

A plethora of software applications has been developed for different steps in the workflow, from data acquisition, through data processing to statistical or machine learning analysis, and subsequent interpretation (Chang et al. 2021; Misra 2021), and the specific software used should be reported.

### 16. Strategies to avoid false positives

Where applicable, describe what methods were used to correct for multiple testing and to determine significance.

#### Example:

“Statistical significance was defined as a P value less than a Bonferroni-corrected threshold adjusting for the number of metabolites or metabolite ratios available at the applicable visit. ….. To provide information related to false discovery rate, Q values were additionally estimated from the P values obtained for the cross-sectional analyses using the R package QVALUE.

(Sekula et al. 2016)

#### Example:

“To take into account the high degree of correlation in spectral data, we used a permutation-based method to estimate across the three cohort studies the the Metabolome Wide Significance Level (MWSL or α’). For each permutation, we randomly allocate the outcome of interest to each study participant across cohort studies to mimic the null hypothesis of no association; we then calculate the P-value for each spectral variable using a linear regression model as described above and record the lowest P-value. We ran 10 000 permutations per outcome (CAC or IMT) for both 1D NMR and CPMG spectra. The per-variable significance level α’ giving a 5% Family-Wise Error Rate (FWER, α) corresponds to the 500^th^ of these lowest P-values. The effective number of tests (ENT) is defined as the number of independent tests that would be required to obtain the same significance level using Bonferroni correction: ENT = α/α’. We computed P-value thresholds for each combination of model, outcome, and ^1^H NMR assay type….and defined a unified threshold derived from the median ENT for both 1D NMR and CPMG”

(Tzoulaki et al. 2019)

**Explanation:** Because metabolomic data are high dimensional, many hundreds or thousands of hypothesis tests may be conducted simultaneously in a metabolome wide association study. Consequently, it is frequently necessary to adjust for multiple testing to minimize the risk of false positive associations (Peluso, Glen, and Ebbels 2021). The choice of methods for adjustment and selection of a suitable significance threshold is complicated in metabolomics by the highly collinear nature of the metabolome, the existence of peak overlap within metabolic spectral data, the existence of metabolites in co-regulated and redundant biological pathways, and the non-normality of metabolite levels (Chadeau-Hyam et al. 2010). Unlike in some other omic technologies, a gold standard approach for dealing with multiple testing has, therefore, not yet been established. Most, commonly applied, false discovery methods assume independence of metabolites and only correct for Type 1 errors. Cross-validation approaches are another means of addressing false positives. Consequently, it is vital that studies are transparent in the reporting of how they chose to define significance and deal with multiple testing and justify their choice, as this can have important implications for the assessment of their ‘significant’ findings.

### 17. Independent validation

Describe if and how independent validation of findings was attempted. How were findings chosen for validation, what criteria were used to determine validation. If an independent population was used, this should also be described in Study setting and participants.

#### Example:

“total of 263 compounds (including targeted and nontargeted) that were significantly associated with incident diabetes in JHS model 1 and 107 compounds from JHS model 2 were measured in MESA and were nominated for replication. …. Model discrimination was validated in MESA. The same prediction models were used except for the exclusion of parental history of diabetes due to data availability.”

#### Example:

“The models fitted in the UK Biobank were exported via ONNX, and calculation of metabolomic states was replicated in the Whitehall II Cohort, the Rotterdam Study, the Leiden Longevity Study and the PROspective Study of Pravastatin in the Elderly at Risk”

(Buergel et al. 2022)

**Explanation:** Despite a plethora of significant findings, few potential biomarkers identified in metabolomic epidemiology studies have progressed to the clinic (Li et al. 2024). One of the primary reasons for this is the lack of validation of these findings in suitable independent populations (Perng and Aslibekyan 2020). While via cross-validation or permutation testing within the same dataset are often employed to determine model performance, they are susceptible to overfitting and do not account for population level differences which could affect relationships between a metabolite and a phenotype or exposure. Ideally, the performance of the model should be evaluated on independent data (Lopez-Lopez et al. 2018). Therefore, it should be reported whether external/independent validation of significant findings was attempted. In order for the reader to understand whether a lack of validation may be due to systematic differences between the discovery and validation populations, such as ethnicity, sociodemographic characteristics, recruitment or sampling strategy, ascertainment of key variables, or time-point of investigation, lifestyle and environmental factors, as opposed to false positives (Perng and Aslibekyan 2020), the demographics and study characteristics of the validation population should also be reported. Finally, it should be stated how validation will be determined, for example via significance threshold, effect size and direction of effect. For a detailed explanation of validation versus replication in metabolomics studies, see Perng & Asilbekyan (Perng and Aslibekyan 2020).

### 18. Pathway analysis/pathway enrichment/Metabolite set analysis

- Report the pathway analysis method used, software package, version and values of associated parameters as well as the pathway database used, release number, compound identifier type, and which organism-specific pathway set was used.
- Multiple testing correction approach and associated adjusted significance level should be stated.
- If applying Over Representation Analysis, report background metabolite set and statistical method for selection of significant metabolites.

#### Example:

“The data input files were uploaded to the MetaboAnalyst 5.0 web server, where data pre-processing, such as normalization and scaling, and metabolic pathway analysis were performed. Then, we specified pathway analysis parameters as follows: [1] global test method performed enrichment analysis, [2] Relative Betweenness was used to measure centrality, and [3] 80 human metabolic pathways (Homo sapiens) in the Kyoto Encyclopedia of Genes and Genomes (KEGG) database were employed as reference metabolic pathways. The pathway enrichment and topological analysis algorithms were previously illustrated……..MetabolomicsWorkbench RefMet matched each metabolite with its corresponding subclass. MetabolomicsWorkbench RefMet is a standardized reference terminology for metabolomics. RefMet is essential for comparing and contrasting metabolite data across different experiments and studies”.

(Banimfreg et al. 2022)

#### Example:

“The ORs and unadjusted P-values of the lipid species were used as input for the chemical similarity enrichment analysis using ChemRICH. ChemRICH uses the structure of lipid headgroups and the degree of saturation in acyl chains [saturated fatty acids (SFA)=0, monounsaturated fatty acids (MUFA)=1, and polyunsaturated fatty acids (PUFA)≥2] to cluster lipids into non-overlapping chemical groups. At least three lipids were required to form a cluster. Lipids are poorly represented in canonical biochemical pathway databases, so a traditional pathway analysis approach is prone to ignore many lipid classes with several statistically significant lipid species. Therefore, we chose a lipid set enrichment analysis using the ChemRICH software to identify the significant lipid classes that were associated with liver cancer risk. ChemRICH compares the observed p-value distribution for a lipid set against a uniform p-value distribution with an underlying hypothesis that a non-significant class will follow a uniform p-value distribution. ChemRICH uses a Kolmogorov–Smirnov (KS) test to compare these p-value distributions and does not rely on a background database, thus ChemRICH includes all the annotated lipids in the set analysis. Using this reference distribution, KS tests were used to determine lipid clusters where P-values showed sufficient evidence of departure from the null distribution. ChemRICH P-values were adjusted using the Benjamini-Hochberg (BH) procedure for false discovery rate control at level 0.05.”

(Barupal et al. 2024)

**Explanation:** Enrichment approaches can provide a more global understanding of the biological disruption that accompanies the exposure or outcome of interest, and multiple different approaches for conducting such analyses have been developed (Lu, Pang, and Xia 2023). While differences between these approaches mean the exact items to be reported differ, in general, the approach taken, the software used, the pathway reference library, multiple testing correction and any other pertinent parameters should be reported. Any study using over representation analysis should additionally report the background set used as this can critically influence the results. Several excellent reporting guidelines for pathway analyses in metabolomics have been published previously (Wieder et al. 2021).

## RESULTS

### 19. Participants

- Describe the final number(s) of individuals included in the primary analyses
- Provide number excluded with reasons, including, if relevant, those dropped due to missing phenotype data and those dropped due to sample availability/sample failure, insufficient volume etc.
- Use of a flow diagram including all analyses is strongly encouraged.

#### Example:

“We invited 597 women aged 39–64 years, who had undergone a medical examination to participate in this study and obtained informed consent from 526. We also obtained informed consent from 325 patients who had visited our osteoporosis out-patient clinic for the first time. Subjects with the following conditions were excluded (n = 741) from the study: those who had used medications including but not limited to osteoporosis drugs; those with unstable menstruation status; subjects exhibiting a BMI of <18.5 or >30; and individuals with incomplete data sets. Then, the remaining subjects (n = 110) were divided into pre- and post-menopausal groups, and the latter was subdivided into “low BMD” (lower than −1 SD (low estradiol [E2] and low BMD; LL) and “normal BMD” (low E2 and normal BMD; LN) groups. One subject in the pre-menopausal group who showed <20 pg/ml E2 was excluded. Since pre-menopausal individuals exhibited normal bone mass (higher than −1 SD), these individuals were grouped as normal E2 and normal BMD (NN group). The number of subjects in final NN, LL, and LN groups were 30, 46, and 33, respectively”.

(Miyamoto et al. 2018)

#### Example:

**Figure 1:**
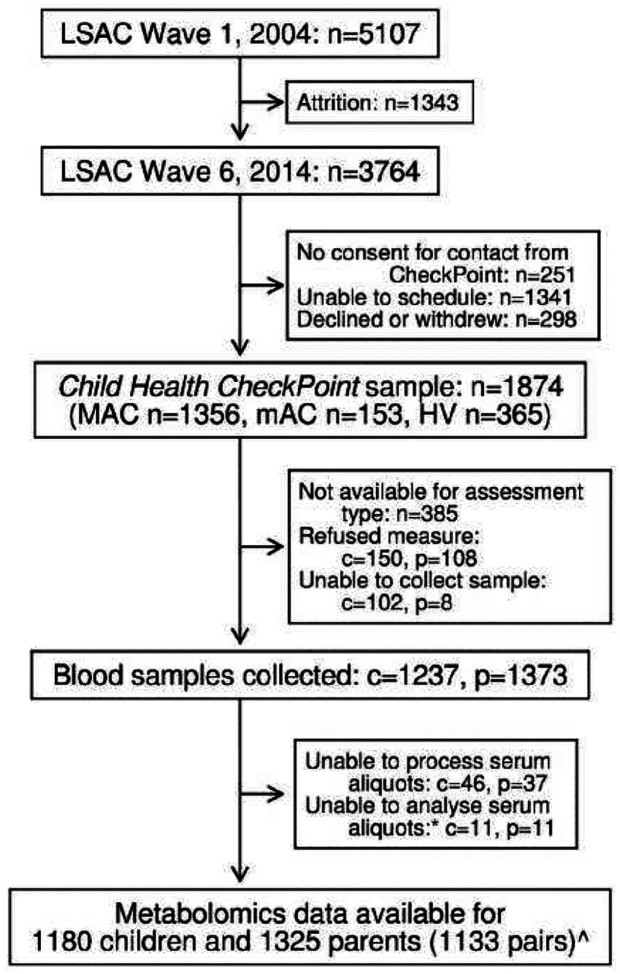
Participant flow chart. *Unable to analyse due to insufficient volume or poor quality sample. Data from six non-biological child–parent pairs excluded from concordance analyses. c, number of children; HV, home visit; LSAC, Longitudinal Study of Australian Children; MAC, main assessment centre; mAC, mini assessment centre; n, number of families; p, number of attending adults. (Ellul et al. 2019)

**Explanation:** It is vital to report the number of participants included in the analyses, particularly for high dimensional global profiling where the number of measured metabolites may exceed the number of participants. Often the number of participants included in the final analyses may differ from the total number who had metabolomic profiling performed. In these cases, the number excluded should be reported and the authors should be transparent in the reasons why. The implications of participants dropped due to lack of relevant phenotype data, differ from those dropped due to issues with the biosamples, and therefore it is important to report. Commonly in metabolomic epidemiology studies stratified and subgroup analyses may be performed, or several endpoints may be analyzed. Authors must also therefore provide the number of participants included in these analyses. A well-designed and structured flow diagram can effectively and succinctly communicate details of the process of enrolling participants that might otherwise necessitate lengthy explanations in the text. Such a diagram may also be beneficial in reporting key findings, for instance, the number of participants meeting criteria for the primary outcome.

### 20. Metabolites

- State number of metabolites/metabolite features reported in the study and whether the values are relative abundance or quantitative.
- Specify the number that were identified/annotated and how many were unknown.
- State the final subset of metabolites that were used in the analysis.

#### Example:

“A detailed list of all analysed metabolites is provided in Table S1. … 148 metabolites were reproducibly measured in serum obtained from the umbilical cord blood from each of the 142 infants studied.

**Table S1:** List of quantified metabolites Included for each metabolite: The recommended limit of detection for the specified platform; percentage of missing values (i.e. metabolite concentration below the measurable limit); mean serum concentration across the whole sample population; The Relative Standard Deviation (RSD) for the two repeat-injection Quality Control patients (8 reps per patient evenly dispersed across the experimental run) – an RSD of < 20% is considered acceptable; The Biological Signal to Noise ratio (S/N) in decibels (dB) – this gives an indication of biological information content, calculated using the following equation: 20 log (RMSsample/RMSQC), where RMS = Root Mean Squared amplitude of the mean centred data. A S/N > 15dB indicates excellent information content.”

idx	Name	Code	Class	Limit of detection (μM)	%age missing	Mean conc. (μM)	%RSD QC1	%RSD QC2	Biological S/N dB‡
1	Carnitine	C0	Acylcarnitines	4	0	14.831	3	2	20.9
2	Decenoylcarnitine	C10:1	Acylcarnitines	0.12	0	0.164	3	4	15.5
3	Dodecanoylcarnitine	C12	Acylcarnitines	0.057	0	0.081	13	5	20.8
4	Dodecanoylcarnitine	C12:1	Acylcarnitines	0.2	0	0.234	3	3	19.2
5	Tetradecanoylcarnitine	C14	Acylcarnitines	0.03	0	0.064	9	13	22.0

NB: Only the first five rows are shown here for demonstration purposes (Walsh et al. 2012)

#### Example:

“A metabolite is described as “putative” following an accurate mass match to the HMDB database. A metabolite is described as “confirmed” following a match to reference standards and/or MS/MS spectrum. A total of 1153 putative metabolites were extracted from the nontargeted metabolomics raw data, of which 959 passed quality control. These putative metabolites were subjected to both sex-combined and sex-stratified comparisons of smokers versus COPD. Of the 959 putative metabolites, 184 were significant at p<0.05 and selected for structural confirmation. Of these 184 metabolites, 67 were structurally confirmed by MS/MS and/or matching to reference standards… All nontargeted metabolomics data presented in this study refer to these 67 structurally confirmed metabolites.”

(Naz et al. 2017)

**Explanation:** There are various methodological approaches in metabolomics. Typically, validated targeted assays will yield quantitative data (concentrations) on a limited number of confidently identified metabolites. Conversely, untargeted data will typically yield hundreds or thousands of metabolites (or features) measured as relative abundance only. Depending on the data processing method chosen, these data might have confident metabolite identities, putatively annotated features, or unannotated features. Untargeted workflows often include strict cleaning steps to ensure that only high-quality data are included in the data analysis. This step can drastically reduce the number of metabolites/features included in the final analysis. It is therefore important to disclose the methodological approaches taken and how these affect the metabolite variables included in the analysis. This level of detail provides a high level of transparency for the readers.

### 21. Descriptive Data

- Provide the primary characteristics of the study participants and information on exposures and potential confounders.
- Indicate number of participants with missing data for the variables of interest.
- For cohort studies summarize how long patients have been followed for, and at what timepoint(s) during the study the metabolomic profiles were measured.

#### Example:

“Our analysis included 110,655 participants without dementia at baseline, with an average (SD) age of 56.5 (8.1), of which 59,469 (53.7%) were female. After a median follow-up of 12.2 years (IQR: 11.5–12.9 years), 1439 (1.30%) participants developed dementia. Baseline characteristics of all participants stratified by incident dementia are summarized in Table1.1. Participants with incident dementia were often older, male, APOE ε4 carriers, with lower education, higher systolic pressures, a history of diabetes mellitus, anti-hypertensive medication use, former or current smokers, and had a history of stroke or coronary heart disease”.

(Zhang et al. 2022)

#### Example:

“We extracted clinical predictors and disease endpoints for 117,981 individuals with serum NMR profiling at the time of cohort recruitment …. The study population had a median age of 58 years (interquartile range (IQR) 50, 63), of whom 54.2% were female, 11% current smokers and 5.2% diagnosed with T2D (Table 1). Median body mass index (BMI) was 26.8 (IQR 24.2, 29.9), systolic blood pressure was 136 mmHg (IQR 124, 149), total cholesterol was 5.65 mmol l^−1^ (IQR 4.90, 6.42) and glucose was 4.93 mmol l^−1^ (IQR 4.60, 5.32). Median follow-up was 12.2 years with ….1,435,340 overall person-years”.

(Buergel et al. 2022)

#### Example:

“We analyzed data from 118,021 participants from UK Biobank with metabolomics measurements, of whom 54% were female and 95% were White, with a mean age of 56.5 (SD: 8.1) years. The median follow-up time was 13.1 years (5th-95th percentile, 11.8–14.4 years). The characteristics of UK Biobank participants whose blood samples were randomly selected for the metabolomics assay to be performed were broadly comparable with those who were not selected”

(Harshfield and Markus 2023)

**Explanation:** To assess the generalizability of the findings, summary statistics; mean, range, standard deviation, proportion as appropriate must be provided on participants characteristics. These should include demographic factors such as sex and age, clinical factors such as BMI and disease state, and social factors such as SES and geographical location/setting, as appropriate. These should also include any potential confounding variables that do not fall into any of these categories, for example if a metabolomic epidemiology study is nested within a trial or intervention study, the authors should also provide information on how the participants were assigned. Increasingly, metabolomic studies are being conducted within the context of existing biobanks, in which case this should also be stated. For exposures of interests the authors should also provide information on levels in the participants included in the analyses. Together, this information will allow the reader to understand the population in which the results were generated and assess how applicable they are to other populations and settings. The information can be provided in the text and/or a table/figure and should explicitly state for each of the variables whether any information is missing for any of these participants. For cohort and longitudinal studies, information must be provided on the length of follow up and at what timepoint(s) during the study the metabolomic profiles were measured. A study schematic figure can be very helpful in this regard.

### 22. Main Results (Single Metabolite Models)

- Provide unadjusted effect estimates and, if applicable, confounder-adjusted estimates and their precision (e.g., 95% confidence interval). State which confounders were adjusted for.
- Report results that were robust to correction for multiple testing,
- Sensitivity analyses and related approaches to corroborate findings and strengthen causal inference
- If multiple statistical methods have been used, report approach used to define consensus.

#### Example:

“Three types of multivariable linear regression models (unstratified, infant sex-stratified, and maternal pre-pregnancy weight status-stratified) were evaluated. Figure 1 presents the Manhattan plots for the metabolites that showed statistical significance after an FDR correction (qvalue < 0.05) from the adjusted linear regression models. Table 2 presents the summary of the metabolites that had significant associations with each outcome after the multiple testing correction. Twenty-three metabolites/CMI measured in the infant cord blood samples were associated (q value < 0.05) with birth weight Z-score. Thirteen of these metabolites had a negative association with birth weight Z-score including 11 AC, 1 lysoPC, and 1 CMI, the ratio of polyunsaturated fatty acid to monounsaturated fatty acid. Ten metabolites with a positive association with birth weight Z-score included 7 lysoPCs and 3 CMIs (the ratios of total lysophosphatidylcholine to total phosphatidylcholine, monounsaturated fatty acid to saturated fatty acid, and total lysophosphatidylcholine. Six metabolites measured in the infant cord blood samples were associated (q value < 0.05) with birth length Z-score. One AC was negatively associated, and 5 metabolites (4 lysoPCs and 1 CMI, the ratio of Total to over total phosphatidylcholine) were positively associated with birth length Z-score. No statistically significant associations were found between cord plasma metabolites and birth weight-for-length Z-score.”

(Yeum et al. 2023)

#### Example:

“We analyzed the association of 325 baseline metabolic measures with MRI markers at the first imaging assessment in cross-sectional analyses adjusted for age at recruitment and sex ….. Lower levels of apolipoproteins, cholesterol, free cholesterol, cholesteryl esters, fatty acids, lipoprotein particle concentrations, phospholipids, triglycerides, and total lipids and higher levels of amino acids, glucose, and glycoprotein acetyls (GlycA; an inflammatory marker) were associated with increased white matter microstructural damage on DTI, as indicated by higher WMH and MD and lower FA. In cross-sectional analyses adjusted for possible confounders and additional vascular risk factors, most metabolites remained significantly associated with FA and MD …… Lower levels of apolipoproteins, cholesterol, free cholesterol, cholesteryl esters, fatty acids, lipoprotein particle concentrations, phospholipids, triglycerides, and total lipids were associated with higher MD and lower FA on DTI, indicating increased white matter microstructural damage. However, increased total lipids in very large HDL (XL_HDL_L) and increased HDL particle size (HDL_size) were associated with lower FA (OR: 1.44, 95% CI 1.07–1.95, and OR: 1.19, 95% CI 1.06–1.34, respectively) and higher MD (OR: 1.49, 95% CI 1.11–2.01, and OR: 1.24, 95% CI 1.11–1.40, respectively). Higher levels of high-density lipoprotein cholesterol (HCL_C) and lipoproteins within large HDL and very large HDL were also associated with increased white matter microstructural damage”.

(Harshfield and Markus 2023)

#### Example:

“The associations between plasma glucose and the discriminant phenolic compounds according to the three adjustment models are presented in Table 3. Dihydrocaffeic acid showed a significant inverse association with plasma glucose in model 2 (β = − 17.12 (95 % CI: − 29.92; − 4.32) mg/dL per 1-SD, p-value = 0.009), but in model 3, which included the use of antidiabetic drugs, the relationship was no longer significant and the β coefficient decreased. However, a strong positive correlation was found between antidiabetic drug usage and plasma glucose (data not shown), so its inclusion as a confounder could be an overadjustment. A comparison of the associations of dihydrocaffeic acid with plasma glucose using adjustment models 2 and 3 is presented in Fig. 3. Genistein diglucuronide did not show any significant association in any adjustment model”

(Dominguez-Lopez et al. 2023).

**Explanation:** In many instances, such as studies seeking biomarkers or those attempting to replicate previous findings, the single metabolite models will be the key results of interest. We note for such models, metabolites can represent either the exposure or the outcome depending on the research question of interest. In both cases, the authors should state what other covariates are being adjusted for in their models, where more than one set of covariates were adjusted for in different models, the results from all models should be provided and differences in results between the models noted. Whether these results were robust to correction for multiple testing should also be explicitly stated. All results regardless of significance should be presented somewhere in the manuscript (including as supplementary tables and figures) for the purposes of future meta-analysis or replication attempts. Associated uncertainty metrics around the effect estimates should be reported for the same reason. A correlation matrix of abundances between the significant metabolites may also be of use as an indicator of potential correlation between significant findings.

### 23. Main Results (Multiple Metabolite Models)

- Report type of model and its main parameters, including whether the approach is supervised/unsupervised
- Report model validation statistics (cross validation, permutation etc.)
- Report main findings: how well does the model predict the outcome in supervised approaches (with associated statistical uncertainty metrics) and/or how important features were identified in the model (with associated statistical uncertainty metrics)

#### Example:

**“**We proceeded to construct models predicting obesity from the lipidome of the FINRISK data set. Models were trained on lipid subspecies, including age and sex as covariables. Using a Lasso model trained in a cross-validation loop, we first used BMI as our obesity measure and reached a mean absolute error (MAE) of 2.5 ± 0.18 and an explained variation of 47%. Then, using the same procedure, we analyzed how the plasma lipidome is predicting other obesity measures compared with the models we obtained for BMI using a normalized MAE. On this comparable metric, BMI was outperformed by WC (MAE = 6.5 ± 0.59, 64% variation explained, WHR (MAE = 0.039 ± 0.0033, 65% variation explained), and BFP (MAE = 3.6 ± 0.33, 73% variation explained).

…

Training of the BFP model on the FINRISK test set resulted in a cross-validation MAE of 3.61 ± 0.33 BFP units, which is about 8% of the BFP range. The training error of the model was found at a MAE of 3.33 BFP units, and the mean error of the hold out FINRISK test data was at 3.84. We validated the FINRISK based BFP model in a second, independent data set (MDC-CC), the clinical baseline characteristics of which differ from the FINRISK data set.

This validation resulted in a MAE of 3.67, which is only slightly above the cross-validation error obtained with the FINRISK data set. The validation also confirms that the models obtained were independent of the fasting duration, because the participants from the MDC-CC cohort were fasted over night.”

(Gerl et al. 2019)

#### Example:

“based on the 33 identified differential metabolites in LA (n = 136) vs BN (n = 170), an optimal variable combination of biomarkers for nodule classification were obtained by least absolute shrinkage and selection operator (LASSO)- binary logistic regression model. A ten-fold cross validation was used to test the robustness of the model. Variable selection and parameter regularization were adjusted by the penalization in likelihood maximization through parameter λ^24^. The global metabolomic analysis was further performed independently in an internal validation (n = 104) and an external validation (n = 111) cohort to verify the classification performance of the discriminant model. As a result, a panel of 27 metabolites was identified in the discovery set as the best discriminant model with maximum of the mean AUC, including 9 upregulated and 18 downregulated in LA compared with BN.”

(Yao et al. 2023)

**Explanation:** If using an analysis technique which models several metabolite variables at a time (e.g. multivariate regression or machine learning models) the results will need to be described in more detail than for a traditional linear model. It is helpful to place the approach into a general framework (e.g. supervised learning) and to refer to other sources for methodological detail. Unlike simple linear models, quantitative evidence that overfitting has been avoided is critical (e.g. cross validation statistics), as is the reporting of which metabolites were selected as the most important together with their associated metrics (e.g. empirical p-values from permutation procedures). If a summary score representing multiple metabolites was created, the association of this score with the exposure/outcome should be reported together with the statistical uncertainty metrics. The contribution of each metabolite to the score should also be provided. For machine learning, the approach to train/test splitting should be stated. If a discovery and validation cohorts were used, the results for both should be reported.

### 24. Pathway Analysis

- Report the coverage of pathways (number of quantified metabolites relative to the size of the pathway, e.g. min, median, max).
- Report number and identity of pathways that are enriched and the associated metrics to describe their enrichment.

#### Example:

**Figure 2:**
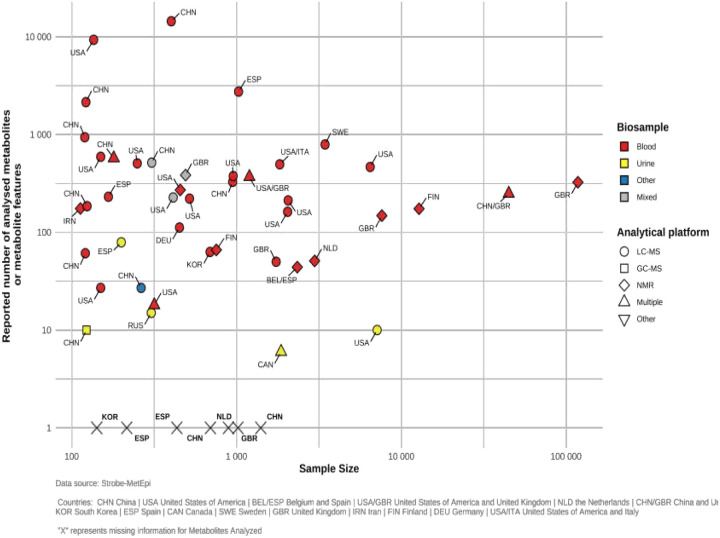
Characteristics of the 50 included human metabolomic studies. Each point represents one of the 50 included studies, with sample size plotted against the number of metabolites or metabolite features reported. The axes are plotted on the logarithmic scale to account for the large range. The sample size (x-axis) is plotted against the number of metabolites or metabolite features reported (y-axis). Sample sizes ranged from 113 to 118,021 participants and the number of metabolites from 6 to 14,414. Both axes are plotted on the logarithmic scale to account for the large range. Point color indicates the type of biosample, and the point shape denotes the analytical platform used for metabolite measurement. Country refers to the population in which the analysis was conducted.

“The MetaboAnalyst 5.0 platform was used to perform pathway enrichment and topological analysis of differential metabolites in blood. Groupwise pathway analyses were performed assuming the three groups indicated in Fig. 2B (non-diabetics, prediabetics, and controlled diabetics, and uncontrolled diabetics). Based on the identified metabolites in our data, the MetaboAnalyst 5.0 platform detected 39 metabolic pathways for each grouped comparison mentioned above. These metabolic pathways are shown in Fig. 6. Potential target pathways were screened according to each grouped comparison’s -log(P) value and pathway impact score. The arbitrary FDR cut-off values (fdrcut-off) are 0.00007, 0.000038, and 0.0417 for non-diabetics vs. uncontrolled diabetics, non-diabetics vs. prediabetics and controlled diabetics, and uncontrolled diabetics vs. prediabetics and controlled diabetics, respectively. However, there was no considerable increase in significantly enriched pathways when the fdrcut-off was increased beyond the preferred values. Therefore, including all pathways would lead to a slight change in the results. Thus, the number of selected pathways was estimated based on each grouped comparison outcome”

(Banimfreg et al. 2022)

#### Example:

**Figure 3:**
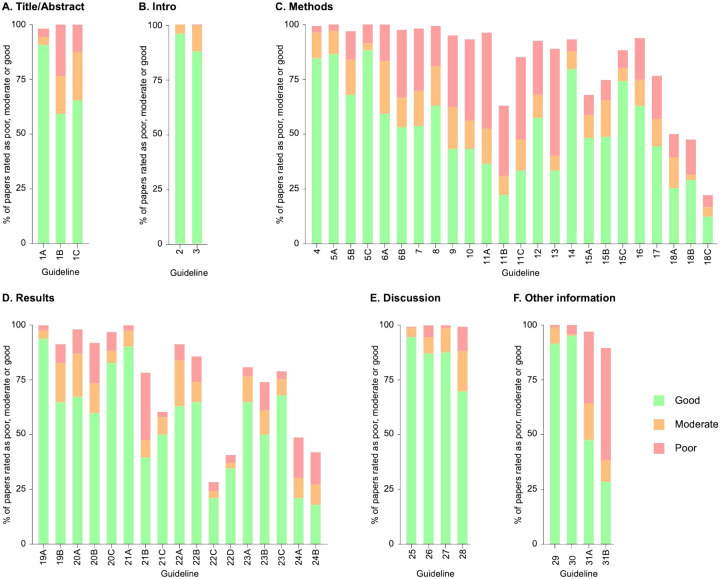
Performance of each checklist item in the 50 included human metabolomic studies. For each item (1A-32 on the x-axis), the stacked bars show the percentage of papers rated as good (dark), moderate (medium), or poor (light) by reviewers, averaged across all reviewers for that paper. Where the stacked bars do not sum to 100%, the remainder reflects papers rated as not applicable for that item. Each item was assessed up to 162 times across the 50 studies and 16 reviewers (each reviewer assessed 3 or 4 papers). Across all reviewers and all papers each checklist item was reviewed 162 times (16 reviewers reviewed 3 or 4 papers and there were 50 papers in total) For each checklist item percentage for each category (good, moderate or poor) was calculated by (Count of that category/162)*100 Where a given checklist item totals <100% across the three categories, this indicates that reviewers ranked some papers as “not applicable” for this category

“POAG is multifactorial and clinically heterogeneous. Investigating heterogeneity in VF loss patterns for POAG may provide new etiologic insights as different types of optic nerve damage manifest as distinct VF loss patterns. For example, glaucomatous paracentral scotomas have been associated with more systemic risk factors compared to peripheral VF loss^21–23^. Therefore, we separately evaluated the associations between metabolite classes and POAG defined by VF loss patterns (paracentral (Fig. 3a) versus peripheral VF loss (Fig. 3b)). Of the 599 cases, VF loss patterns derived from Humphrey visual field test were available in 509 cases. As shown in Fig. 3a and Fig. 3b, overall, more metabolite classes (9 classes versus 5 classes) were significantly associated with POAG with paracentral VF loss versus peripheral VF loss. In addition to the three classes associated with peripheral VF loss, namely DGs, LPCs, and LPEs, for POAG with paracentral VF loss, the classes of TGs, PEs, and PCs were also adversely associated at FDR < 0.05 (Model 5). Notably, the adverse relation between TGs/DGs/PEs and POAG with paracentral VF loss was significant at the FDR < 0.001 level. Furthermore, in addition to carnitines and organic acids and derivatives being inversely associated with POAG with peripheral VF loss, cholesteryl esters were also inversely associated with POAG with paracentral VF loss at FDR < 0.05 (Model 5).”

(Zeleznik et al. 2023)

#### Example:

“To identify key enriched metabolic pathways, we performed MetaboAnalyst-based pathway enrichment analysis on the differentiated metabolites in PF and serum…. the urea cycle, betaine metabolism, and multiple amino acid metabolism pathways (eg, glycine and serine metabolism, arginine and proline metabolism, aspartate metabolism, methionine metabolism) were significantly enriched in PF signatures that differentiated POAF from non-POAF. In regard to the signatures in serum the urea cycle and several amino acid metabolism pathways (such as tyrosine metabolism, arginine and proline metabolism, glycine and serine metabolism, and aspartate metabolism) were also enriched. In addition, several lipid metabolism-related pathways were also enriched in PF and serum signatures, such as carnitine synthesis, phosphatidylinositol phosphate metabolism, and mitochondrial fatty acid metabolism. The interaction network among the enriched metabolic pathways …. demonstrated that the enriched metabolic pathways in PF showed stronger interactions with each other than those in serum, indicating strongly coordinated effects for the metabolic changes in the PF microenvironment”

(Yang et al. 2023)

**Explanation:** Pathway analysis is a very common task in the latter stages of an analysis pipeline. However, with metabolite data it can be easy to make errors or over interpret results, especially when using methods built for other types of data (e.g. transcriptomics). Key aspects to consider are the effects of incomplete metabolite annotations, low coverage of many pathways, inconsistent pathway definitions between pathway databases, default background lists and effect of multiple testing corrections which can all have a huge effect on results (Wieder et al. 2021). Best practice will be to repeat the analysis using at least 2 different pathway databases. The authors should report issues of low coverage, uncertainty in annotations and differences between pathway databases. None of the examples here exhibit full best practice reporting. We refer readers to two recent discussions on the limitations of pathway analysis in metabolomics (Lee, Su, and Huan 2025; Tsouka and Masoodi 2023)

## DISCUSSION

### 25. Key Results:

Summarize key results with reference to study objectives

#### Example:

“Metabolomic profiling of statin use in longitudinal cohorts uncovered an intricate association pattern of circulating lipoprotein, fatty acid, and metabolite changes, which adds to our understanding of the LDL-C–independent effects of statins. Statin use was associated with pronounced lowering of numerous lipids and fatty acids consistent with the cardioprotective effects. By contrast, statin use did not markedly affect the circulating levels of recently identified biomarkers for cardiometabolic risk such as amino acids, glycolysis- and glycogenesis-related metabolites, or ketone bodies.”

(Wurtz et al. 2016)

#### Example:

“Using an untargeted metabolomics platform and a comprehensive pathway analysis tool in a large, multiethnic population cohort (MESA), we identified a metabolite signature associated with VAT linked to several putative biological pathways, including amino acid substrate use/metabolism and glycolysis/gluconeogenesis. We then replicated our findings in a separate epidemiological cohort (NEO study) using targeted metabolomics and found that acetylglycoproteins, branched-chain amino acids (isoleucine, leucine, and valine), glutamine (inversely), and serum triglycerides by ^1^H NMR remained associated with VAT, even after adjustment for established surrogate biomarkers of VAT (BMI, fasting glucose, waist circumference, and serum triglycerides), suggesting that a single, fasting measurement of metabolites can provide biological information beyond standard risk markers of visceral fat.”

(Neeland et al. 2019)

#### Example:

“In older people with T2D, a metabolomic risk score (which integrated information on lipoproteins, amino acids, ketone bodies, and markers of fluid balance) provided some improvement in the prediction of 10-year CV risk over and above traditional CV risk factors. Slight improvement was seen in predictive performance of the updated model with the MRS in terms of discrimination and calibration, and there was better reclassification, in comparison with the reference model based on QRISK3.”

(Huang et al. 2023)

**Explanation:** It is good practice to begin the discussion with a short summary of the main findings of the study, which will help readers to assess whether the subsequent interpretation and implications offered by the authors are supported by the findings. The summary should be put in perspective with the main study objectives and focus on any prespecified hypotheses.

### 26. Limitations:

Discuss limitations of the study taking into account sources of potential bias or imprecision. Discuss both direction and magnitude of any potential bias.

#### Example:

“Limitations of the metabolomic approach include the large but still restricted number of annotated metabolites, which form a fraction of the incompletely characterised human metabolome. Therefore, metabolites other than tryptophan that are not yet annotated could be better predictors for survival.”

(van Laarhoven et al. 2018)

#### Example:

“Sample handling in the AASK did not follow a standardized protocol and the storage period was many years. As a result, some metabolites may have been missed, but those that were identified are likely to be robust to a range of handling techniques and long-term storage. The identity of some of the most strongly correlated metabolites with mGFR was unknown (…). GFR measurement is known to be imprecise, but this inflates the reported GFR estimation errors. Furthermore, iothalamate and iohexol GFR measurement methods differ systematically and standardization of GFR for BSA may not optimally deal with variation in body composition.”

(Coresh et al. 2019)

#### Example:

“Preanalytic factors such as time since last food/drink at blood collection (and time from blood collection to processing in the EPIC-Oxford sub-cohort) contributed to less than 4% to the total variability in the metabolite data. To limit their potential impact on the concentrations of metabolites, pre-analytical factors were adjusted for in the statistical analyses.”

(Carayol et al. 2017)

**Explanation:** Recognizing and examining potential sources of bias is essential when interpreting metabolomic epidemiology studies. The recognition and examination of the limitations of a research study are crucial components of scientific reporting. Metabolomic epidemiology studies should report on those limitations that relate to epidemiology, such as the overarching study design (Vandenbroucke et al. 2007), and those relating to the metabolomics aspects from the collection and handling of samples, the choice of profiling technology to analysis and replication (Tzoulaki et al. 2014; van Roekel et al. 2019). Potential sources of bias, such as toward detecting high-abundance molecules should also be addressed (Turi et al. 2018). Overfitting is a common issue in multivariate analysis, and the inclusion of validation studies is highly desirable. The results of such studies, or their absence, should be addressed.

It is vital not only to highlight the factors that may have influenced the results but also to comment on their importance. This includes considering the probable direction and extent of any potential bias (Petersen et al. 2021). Finally, the authors should acknowledge any lack of precision in the study’s results. This imprecision can manifest in various aspects of the study, such as the sample size and the measurement of exposures, confounders, and outcomes. When the true values of an exposure are measured imprecisely, this will typically lead to a bias towards unity. In simpler terms, the less precise the risk factor measurement, the greater the bias. (von Elm et al. 2007). However, in the context of metabolomic epidemiology, it is essential to note that when highly correlated risk factors are measured with varying degrees of imprecision, the adjusted relative risk associated with them can be biased either towards or away from unity (Phillips and Smith 1991).

### 27. Interpretation:

Give a cautious overall interpretation of results considering objectives, limitations, multiplicity of analyses, results from similar studies, and other relevant evidence. Make biological interpretations, relative to the level of confidence in the metabolite identifications.

#### Example:

“ApoA-1 was more abundant in boys, whereas ApoB was higher in girls, leading to a higher ApoB/ ApoA-1 ratio in girls. The opposite pattern was found in our limited sample of fathers relative to mothers. These data are surprising and differ from a similarly sized study of slightly older European adolescent children (mean age 15 years) that found higher ApoA-1 and ApoB in girls relative to boys. Interestingly, a higher ApoB/ApoA-1 ratio has been strongly linked to increased coronary risk in adults, suggesting that sex differences may alter with increasing age, in keeping with the increased CVD risk in adult men. ApoA-1 is the main protein component of HDL cholesterol thus the differences in trajectories in lipids and HDL cholesterol for boys and girls across childhood that have been reported could partially explain this observation.”

(Ellul et al. 2019)

#### Example:

“…although we used a prospective design in the blood metabolites analysis, the identified associations between metabolites and Alzheimer’s dementia risk do not necessarily imply causality. It is possible that the relationship between metabolic disturbance and risk for Alzheimer’s dementia is bidirectional. Functional studies or advanced statistical models (e.g., Mendelian randomization) are required to establish the potential causal role of metabolic dysfunction in Alzheimer’s dementia pathology.”

(Huo et al. 2020)

#### Example:

“Dysfunction in glycerophospholipid metabolism is among the most prominent metabolic alterations in cancer, as cancer cells need to continually regulate glycerophospholipids for membrane production, energy acquisition, and molecular signaling. Therefore, it is likely that the observed changes in glycerophospholipid metabolism in this study are the direct consequence of breast cancer and thus suggest disruptions among breast cancer patients”

(Luo et al. 2023).

**Explanation:** Interpreting a study’s findings thoughtfully is central to the discussion section. When interpreting results, authors should consider the design of the study and its objectives, which may range from identifying novel biomarkers, to elucidating disease mechanisms, and developing personalized prevention or treatment strategies. Results should be interpreted taking into account the limitations of the study, the findings from similar studies and other relevant evidence. Interpretation of the biological relevance is often based on mapping metabolites to known metabolic pathways. The biological interpretation should consider the limitations of the databases and approaches to pathway analysis used (Lee, Su, and Huan 2025; Tsouka and Masoodi 2023).

Applying the Bradford Hill causality criteria, (Hill 1965), in metabolomic epidemiology may not be as relevant as in traditional epidemiology. Nonetheless, some aspects of the Bradford Hill criteria, in particular biological causality, but also temporality, consistency, and specificity are often relevant in metabolomic epidemiology. The interpretation of a study’s results may lead to helpful recommendations regarding future research that go beyond “more research is needed” (Vandenbroucke et al. 2007). Following up observed associations in *in vitro* laboratory experiments and animal studies may contribute to support causal relationships in humans. The integration of metabolomic data with other omics data may strengthen causal inference. Mendelian randomization (MR) studies are used to investigate causal relationships between an exposure and an outcome by utilizing genetic variants as instrumental variables (Sanderson et al. 2022). Similarly, causal mediation analysis (VanderWeele 2016) is a statistical technique used to investigate the mechanisms through which an exposure influences an outcome. It can be valuable in the context of metabolomic epidemiology to shed light on the pathways by which metabolites or metabolic profiles may mediate the relationship between an exposure and a health outcome.

### 28. Generalizability:

Discuss the generalizability (external validity) of the study results.

#### Example:

“The AASK [African American Study of Kidney Disease and Hypertension] is a study of African Americans with hypertensive kidney disease and as such is a rather homogeneous population with respect to race, geographical location and diet (US based) and cause of kidney disease. We were able to replicate the findings in US Whites and Blacks with and without kidney disease and thus know that these results are not due to Black ethnicity. However, the relative homogeneity of the samples does not allow us to test the generalizability of the findings.”

(Coresh et al. 2019)

#### Example:

“Our findings from mostly older, non-Hispanic white people might not be generalizable to younger individuals and other race or ethnic groups.”

(Zhang et al. 2025)

**Explanation:** Generalizability, also known as external validity or applicability, refers to the degree to which the findings of a study can be extrapolated or applied to different contexts or situations (Murad et al. 2018). Metabolomic associations with an exposure or phenotype can vary across different populations due to genetic, environmental, and lifestyle factors. Consequently, findings from metabolomic epidemiological studies conducted in one population may not directly apply to other populations. When assessing the generalizability of metabolomic epidemiology studies, authors should consider the eligibility criteria for inclusion into the study and the representativeness of the study population. If the study included a specific subgroup or used matching to make groups comparable, generalizing the findings to broader populations may be challenging. Replication of findings in diverse independent populations is important to establish the generalizability of metabolomic associations. Validation studies help confirm the robustness and reproducibility of associations between metabolites and health outcomes across different populations, increasing the confidence in their general applicability. Adjusting for potential confounding factors and conducting sensitivity analyses can also help strengthen the generalizability of results.

Assessing the external validity of study results typically requires judgment. It is crucial for authors to provide adequate information about the study’s context, locations, eligibility criteria, measurement of exposures, outcome definitions, recruitment period, and follow-up duration to assist readers in making such judgments (Vandenbroucke et al. 2007).

## OTHER INFORMATION

### 29. Funding

Describe sources of funding and the role of funders in the present study and, if applicable, sources of funding for the databases and original study or studies on which the present study is based.

#### Example:

“The UK Medical Research Council and Wellcome Trust (Grant: 102215/2/13/2) and the University of Bristol provide core support for ALSPAC. Data collection and metabolic phenotyping in the ALSPAC mother’s study were obtained from British Heart Foundation (SP/07/008/24066) and the Wellcome Trust (WT092830M).”

(Wurtz et al. 2016)

#### Example:

“This work was supported in part by research funds from the Yamagata Prefectural Government and the city of Tsuruoka and by the Grant-in-Aid for Scientific Research (grant numbers 24390168 and 15H04778) from the Japan Society for the Promotion of Science. The funders had no role in study design, data collection and analysis, decision to publish, or preparation of the manuscript.”

(Fukai et al. 2016)

**Explanation:** Most journals require authors to disclose the presence or absence of financial and other conflicts of interest. Several studies have shown a correlation between the source of funding and the conclusions of research articles. For example, the conclusions of clinical trials were more likely to recommend the trial drug as the drug of choice if the trial was funded by for-profit organizations with an interest in the drug (Lexchin et al. 2003). Authors or funders may have conflicts of interest that influence: study design, choice of exposures, outcomes, statistical methods, and selective publication of results (Catalogue of Bias Collaboration 2019). Consequently, the role of the funders should be described in detail including for which part of the study they were directly responsible if applicable (e.g. design, data collection, analysis, writing of the manuscript, decision to publish) (Vandenbroucke et al. 2007).

### 30. Conflicts of Interest.

State whether any of the authors have any conflicts of interest relevant to the proposed work.

#### Example:

“M.S. is a co-founder and member of the scientific advisory boards of the following: Personalis, SensOmics, Filtricine, Qbio, January, Mirvie, and Oralome. He is a member of the scientific advisory board of Jungla. M.M. is a co-founder of Mirvie. L.L., M.S., and M.M. are inventors on the patent application PCT/US2019/052515 related to this work”

(Liang et al. 2020).

#### Example:

“Würtz and Soininen and Mr. Kangas are shareholders, board members, and employees of Brainshake Ltd., a company offering NMR-based metabolite profiling. Ms. Wang, Dr. Tynkkynen, Dr. Tiainen, and Dr. Kettunen are employees of Brainshake Ltd. Dr. Sattar is a consultant for Amgen, Sanofi, AstraZeneca, and Merck & Co. Dr. Ala-Korpela is a shareholder in Brainshake Ltd. All other authors have reported that they have no relationships relevant to the contents of this paper to disclose”

(Wurtz et al. 2016).

#### Example:

“The authors declare no conflict of interest”.

**Explanation:** Authors should report potential conflicts of interest clearly so that readers can assess whether they may have influenced the study. Sources of undue influence may include employers, such as university administrators for academic researchers or government supervisors, particularly political appointees, for government researchers, as well as advisory committees, litigants, and special interest groups. Commercial involvement may also increase the risk of bias.(Catalogue of Bias Collaboration 2019) Conflicts of interest can undermine trust in research findings among professionals, stakeholders, and the public (Romain 2015). This is particularly important in the context of changing public trust in science since the COVID-19 pandemic (Wong 2024), and the growing commercial value of the metabolomic biomarker market (GlobeNewswire).

### 31. Data and code sharing.

- If the metabolomic data were uploaded to a public repository, provide details of the repository and how these data can be accessed.
- Report whether the code needed to reproduce the results in the article is publicly accessible and if so, where.

#### Example:

“Data are available upon submission and approval of a research proposal to the ALSPAC Executive. Further information can be found at https://proposals.epi.bristol.ac.uk/ and by contacting alspac-exec@bristol.ac.uk”.

(O’Keeffe et al. 2022)

#### Example:

“Mass spectral data are available from MetaboLights under accession number MTBLS702 (https://www.ebi.ac.uk/metabolights/MTBLS702).”

(Kindschuh et al. 2023)

#### Example:

“All software developed for this paper is available at the CIMCB GitHub project page: https://github.com/CIMCB/MetabComparisonBinaryML.”

(Mendez, Reinke, and Broadhurst 2019)

**Explanation:** The rationale for data and code sharing is based on (a) widespread concern about replicability in science (Baker 2016), including metabolomics (Rocca-Serra et al. 2016; Mendez et al. 2019); (b) the potential value of re-analysis using other methods that may yield new biological or methodological insights (Gomes et al. 2022; Witting 2023); (c) the potential value of inclusion in knowledge syntheses with other datasets, e.g. meta-analyses (Gomes et al. 2022; Witting 2023; Spicer and Steinbeck 2018); (d) providing a resource in which questions other than those initially considered can be addressed (Gomes et al. 2022).

Data sharing in metabolomic epidemiology may involve consideration of raw data, transformed data, and annotated data, and also of meta-data (Witting 2023). The complexity of data analysis makes code sharing of different steps in analytical pipelines critically important (Considine and Salek 2019), and can guide replication more precisely than description of analytical steps (Gomes et al. 2022).

Metabolomic data sharing has been promoted by the NIH (https://commonfund.nih.gov/metabolomics), the European Bioinformatics Institute (Haug), *Metabolomics Workbench* (https://www.metabolomicsworkbench.org) and many journals (Spicer and Steinbeck 2018; Haug et al. 2013; Haug, Salek, and Steinbeck 2017). We also suggest authors refer to the FAIR (Findability, Accessibility, Interoperability, and Reusability) guidelines when considering the deposition of their data (Wilkinson et al. 2016).

### Concluding Remarks

Across the flow of a metabolomic epidemiology study the research team is continually choosing and refining approaches and parameters, making each study unique (Playdon et al. 2019). The STROBE-MetEpi Statement aims to act as a resource for authors publishing metabolomics studies. It encourages the most comprehensive and transparent reporting of all stages of their study: what was planned, what was done, and what was found. This will enable the reader to assess the strengths, limitations, and generalizability of a study and to interpret its results in context. Further, it will ensure that the reporting of methods is complete enough to allow for replication of findings of interest. Although this is not its primary use, consulting the guidelines may also be advantageous during the planning of a study, particularly for those new to the field. Through this elaboration and explanation document, we aim to explain why we consider the included checklist items so vital to achieve these aims, as well as demonstrating the need for this checklist via a review of the current literature.

We recognize that metabolomic epidemiology is an evolving field, and therefore we encourage comments, feedback and discussion on the checklist on our website www.strobe-metepi.org to enable its refinement and evolution with the field. Our checklist is also listed on the EQUATOR Network website (https://www.equator-network.org/). We encourage all relevant journals to endorse these guidelines and to recommend the completed checklist be included in supplementary material. We will use our website to monitor uptake and impact of these guidelines with the hope that they can provide a meaningful contribution to the development of standards and the translation of metabolomic data into the public health and clinical spheres.

Transparent reporting will enable the community to understand and interpret these studies within context which will facilitate the replication of key findings, ultimately moving the field toward translation.

## Supplementary Files

This is a list of supplementary files associated with this preprint. Click to download.

- SupplementaryMaterialEnEKellytetalJune26.docx

## Figures and Tables

**Figure 1:**
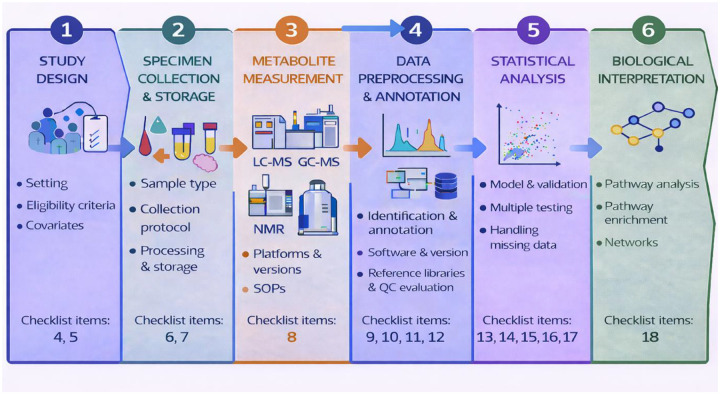
Metabolomic Epidemiology workflow and corresponding checklist items. The figure depicts the key sequential stages in a metabolomic epidemiology study, from study design through to data collection, quality control, statistical analysis, and biological interpretation. The numbered boxes indicate the corresponding STROBE-MetEpi checklist items from the Methods Section

**Table 1.** STROBE-MetEpi checklist of recommended items to address in reports of studies of metabolomic epidemiology. The checklist comprises 31 items organized by section of a scientific paper (Title and Abstract, Introduction, Methods, Results, Discussion, and Other Information). Some items include subitems applicable to specific study designs or analytical platforms. Each item number corresponds to the Explanation and Elaboration section below.

Section	Item	Subitem	Subsection	Recommendation
**TITLE/ ABSTRACT**	1	a	**Title or Abstract: the following information should be provided in the title and/or abstract**	(a) Include the word ‘Metabolomic’, ‘Metabolome’, Metabolite’, or similar
		b		(b) State this is an epidemiological study or use epidemiological terms to make it explicit in the title or abstract. Define the epidemiological study design.
		c		(c) Provide information on analytical technology; biological specimen profiled and size of the study.
**INTRODUCTION**	**2**		**Background**	Explain scientific background and rationale. Justify why metabolomic data are helpful to address the study question(s)
	**3**		**Objectives**	State the overarching objectives. Specify if this a hypothesis-driven or discoverybased/exploratory study, if hypothesis-driven provide the predefined hypothesis. State whether this is a first report or a replication effort for existing results.
**METHODS**	**4**		**Study Design**	Present the key elements of the study design and state whether the study was designed to include metabolomic profiling.
	**5**	**a**	**Study Setting and Participants**	(a) Provide the eligibility and exclusion criteria for study entry, including setting, source population and sampling.
		b		(b) Report on any participant or sample characteristics that could affect the metabolome specifically, if relevant.
		c		(c) Provide details of ethics committee approval and participant informed consent, if relevant.
	**6**	**a**	**Biological Sample Collection, Handling and Storage**	(a) Report on the biological sample type and collection protocol including timing and conditions in sufficient detail to assess quality of sample. Specify whether conditions were the same for every sample in the study.
		b		(b) Report on sample storage prior to processing.
	**7**		**Pre analytical Biological Sample Processing and preparation**	Provide sufficient detail to enable replication of the pre-analytical processing conditions prior to metabolomic profiling. If referencing an existing Standard operation procedure (SOP), provide DOI, version control and any deviations.
	**8**		**Instrumentation and data acquisition**	Provide sufficient detail to enable replication of the analytical method used to collect the metabolomics data, including name and version of instrument, as well as the software version used to control the instrument. If referencing an existing SOP, provide DOI, version control and any deviations.
	**9**		**Data processing**	Provide sufficient detail to enable replication of the transformation of raw data into a data table suitable for analysis, including software package and version number and parameter settings used.
	**10**		**Metabolite identification/annotation**	Describe how metabolite identification or annotation were achieved, including which physicochemical properties the decisions were based on and which reference libraries were used.
	**11**	**a**	**Quality assurance, signal intensity drift correction, quality control, and data cleaning procedures**	(a) Describe all quality assurance, signal intensity drift correction, quality control, and data cleaning procedures; how blanks, system suitability mixes, and quality control samples were prepared and applied before and during data acquisition.
		b		(b) If signal intensity drift correction was performed, reference should be made to the algorithm used.
		c		(c) For quality assessment and data cleaning after data acquisition, include which metrics and/or exclusion criteria were used and how they were applied.
	**12**		**Transformation/ Scaling**	If transformation and scaling were applied prior to statistical analysis, describe which methods were chosen
	**13**		**Handling of missing data**	Describe how missing values were treated for both the metabolomic and covariate data. If imputation was performed, describe the chosen method.
	**14**		**Type of Model**	Provide a clear summary of the model and the framework in which it was performed, specifying dependent and independent variables and co-variates as appropriate.
	**15**	**a**	**Model complexity choice/hyperparameter optimization/model validation**	(a) If relevant, describe the assumptions that pertain to your chosen model and how these assumptions were tested.
		b		(b) Describe how the model was optimized including (as appropriate) formal model selection procedures, model tuning, optimizing number of latent variables, covariate selection; and state why these choices were made.
		c		(c) State the software used, its version, and the procedure applied.
	**16**		**Strategies to avoid false positives**	Where applicable, describe what methods were used to correct for multiple testing and to determine significance.
	**17**		**Independent validation**	Describe if and how validation of findings was attempted. How were findings chosen for validation, what criteria were used to determine validation. If an independent population was used, this should also be described in Study setting and participants.
	**18**	**a**	**Pathway analysis/pathway enrichment/metabolite set analysis**	(a) Report the analysis method used, software package, version and values of associated parameters as well as the database used, release number, compound identifier type, and which organism specific set was used if relevant.
		b		(b) Multiple testing correction approach and associated adjusted significance level should be stated.
		c		(c) If applying Over Representation Analysis, report background metabolite set and statistical method for selection of significant metabolites.
**RESULTS**	**19**	**a**	**Participants**	(a) Describe the final number(s) of individuals included in the primary analyses
		b		(b) Provide number excluded with reasons, including, if relevant, those dropped due to missing phenotype data and those dropped to due to sample availability/sample failure, insufficient volume etc. Use of a flow diagram including all analyses is strongly encouraged.
	**20**	**a**	**Metabolites**	(a) State number of metabolites/metabolite features reported in the study and whether the values are relative abundance or quantitative.
		b		(b) Specify the number that were identified/annotated and how many were unknown.
		c		(c) State the final subset of metabolites that were used in the analysis.
	**21**	**a**	**Descriptive Data**	(a) Provide the primary characteristics of the study participants and information on exposures and potential confounders.
		b		(b) Indicate number of subjects with missing data for the variables of interest.
		c		(c) For cohort studies summarize how long patients have been followed for, and at what timepoint(s) during the study the metabolomic profiles were measured.
	**22**	**a**	**Main Results (Single Metabolite Models)**	(a) Provide unadjusted effect estimates and, if applicable, confounder-adjusted estimates and their precision (e.g., 95% confidence interval). State which confounders were adjusted for.
		b		(b) Report results that were robust to correction for multiple testing, and where relevant those that replicated
		c		(c) Sensitivity analyses and related approaches to corroborate findings and strengthen causal inference
		d		(d) If multiple statistical methods have been used, report approach used to define consensus.
	**23**	**a**	**Main Results (Multiple Metabolite Models)**	(a) Report type of model and its main parameters, including whether the approach is supervised/unsupervised
		b		(b) Report model validation statistics (cross validation, permutation etc.)
		c		(c) Report main findings: how well does the model predict the outcome in supervised approaches (with associated statistical uncertainty metrics) and/or how were important features identified in the model (with associated statistical uncertainty metrics). Which results replicated (if relevant)
	**24**	**a**	**Pathway/Metabolite set Enrichment Analysis**	(a) Report the coverage of metabolite sets (number of quantified metabolites relative to the size of the pathway/set, e.g. min, median, max).
		b		(b) Report number and identity of pathways that are enriched and the associated metrics to describe their enrichment.
**DISCUSSION**	**25**		**Key Results**	Summarize key results with reference to study objectives
	**26**		**Limitations**	Discuss limitations of the study, taking into account sources of potential bias or imprecision. Discuss both direction and magnitude of any potential bias.
	**27**		**Interpretation**	Give a cautious overall interpretation of results considering objectives, limitations, multiplicity of analyses, results from similar studies, and other relevant evidence. Make biological interpretations, relative to the level of confidence in the metabolite identifications.
	**28**		**Generalisability**	Discuss the generalizability (external validity) of the study results.
**OTHER INFORMATION**	**29**		**Funding**	Describe sources of funding and the role of funders in the present study and, if applicable, sources of funding for the databases and original study or studies on which the present study is based.
	**30**	**a**	**Conflicts of interest**	State whether any of the authors have any conflicts of interest relevant to the proposed work.
	**31**	a	** Data and code sharing**	(a) If the metabolomic data were uploaded to a public repository, provide details of the repository and how these data can be accessed.
		**b**		(b) Report whether the code needed to reproduce the results in the article is publicly accessible and, if so, where.

## Box A: The scope of metabolomic epidemiology and STROBE-MetEpi

**Table:**

**Study types addressed**
Case-control studies including Nested-case control and Case-cohort studies
Cohort studies
Cross sectional studies
**Study types not addressed**
Lab reliability/feasibility
Systematic reviews/Literature reviews
Methodological development
Randomized controlled trials
Multi-omic studies

## Box B: Preanalytical Biological Sample Processing and Preparation Details to be Reported

**Table:**

Time between thawing and processing (including time of thawing if applicable).
For solid samples (e.g., tissues) the sample disruption methods employed (e.g., mechanical methods and/or the number and type of freeze-thaw cycles).
For liquid samples (e.g., blood plasma/serum, urine) any protein depletion/crash methods used
Normalization procedures performed to the level of biomass, concentration, or volume
Metabolite sample quenching and extraction methods: Untargeted analysis (e.g., methanol, Folch, Bligh-Dyer) – with information on volume(s) of solvent(s), time of extraction. For biphasic solvents whether the polar on lipophilic phase (or both) is/are analysed. Targeted analysis (quantification) for specific analytes (e.g., methyl-tert-butyl ether (MTBE), chloroform).
For GC-MS, any derivatization methods used (e.g., methoximation, silylation (MSFTA and BSTFA), or fatty acid methyl esterification (FAME)).
For LC-MS, the final solvent in which the sample is reconstituted, including any additional acids or compounds to aid ionization or reagents for chemical ionization.
For quantification of metabolites, provide details as to how this was performed, external or internal calibration with (e.g., isotopic standards, standard addition).
Any storage steps after extraction and before analysis.
Randomization of sample processing order
Preparation of any quality control samples, use of method blanks and order of analyses for the analytical measurements.

## Box C: Optimal Reporting of Instrumentation Settings from Zelena et al. 2009 (Zelena et al. 2009)

**Table S1.** UPLC elution gradient for optimised UPLC-MS methods, (A) ES+ and (B) ES−. A is 99.9/0.1 water/formic acid and B is 99.9/0.1 methanol/formic acid.

A				
Time (min)	Flow Rate (mL/min)	%A	%B	Curve
Initial	0.40	100	0	5
1	0.40	100	0	5
16	0.40	0	100	5
20	0.40	0	100	5
22	0.40	100	0	1

B				
Time (min)	Flow Rate (mL/min)	%A	%B	Curve
Initial	0.36	100	0	4
2	0.36	100	0	4
17	0.36	0	100	4
22	0.36	0	100	4
24	0.36	100	0	1

**Table S2.** Time of Flight mass spectrometer instrumental settings for optimised UPLC-ToF-MS methods in ESI+ and ESI−.

File Type	parameters	ESI positive ion mode	ESI negative ion mode
Scan Settings	mass range (Da)	50–1000	50–1000
	mode	V	V
	data format	centroid	centroid
	DRE	on	on
	scan time (seconds)	0.4	0.4
	lockmass scan frequency (scans)	25	25
	lock mass normal	^13^C peak of LE	^13^C peak of LE
	lock mass attenuated	^12^C peak of LE	^12^C peak of LE
	scans to average	5	5
LC settings	flow rate (mL/min)	0.40	0.36
	column temperature (°C)	50	50
	autosampler temperature (°C)	4	4
	injection mode	partial loop with needle overfill	partial loop with needle overfill
MS settings	capillary voltage (V)	1600	2000
	sample cone voltage (V)	10	10
	desolvation temperature (°C)	480	430
	source temperature (°C)	140	140
	cone gas flow (L.h^−1^)	10	10
	desolvation gas flow (L.h^−1^)	800	800
	MCP detector voltage (V)	2300	2300
	acquisition setup: centroid threshold	1	1
	acquisition setup: minimum points	5	5
	acquisition setup: Np multiplier	0.7	0.7
	TDC settings: trigger threshold (mV)	600	600
	TDC settings: signal threshold (mV)	40	40
	Ion guide 1 initial and final RF voltage (V)	120	170
	Ion guide 2 initial and final RF voltage (V)	240	250
	Hexapole RF voltage (V)	120	170

## Box D: Example of reported metabolite annotation

**Table:**

Metabolite	InChIKey	HMDB ID	SMILES	Ion	Detected *m/z*	*m/z* error (ppm)	Detected RT (min)	RT deviation (min)	MS/MS dot product similarity score
Phenylalanine	COLNVL DHVKW LRT-UHFFFA OYSA-N	HMDB0000159	C1=CC=C(C=C1)C[C@@H](C(=O)O)N	[M+H]+	166.08683	3.4	4.2	0.2	884
Tryptophan	QIVBCDI JIAJPQS-UHFFFA OYSA-N	HMDB0000929	C1=CC=C2C(=C1)C(=CN2)C[C@@H](C(=O)O)N	[M-H]−	203.08303	2.1	4.5	−0.1	687
Valine	KZSNJW FQEVHD MF-UHFFFA OYSA-N	HMDB0000883	CC(C)[C@@H](C(=O)O)N	[M+C2H2O2+H]+	72.08020	−8.0	5.0	0.1	NA

InChIKey – hashed version of the International Chemical Identifiers, developed by the International Union of Pure and Applied Chemistry (IUPAC). They are a text representation of molecule information describing the various properties of the molecule. including the chemical formula, atom connections and hydrogen atoms, charge, stereochemical and isotopic information.

HMDB- Human Metabolome Database

SMILES - Simplified Molecular Input Line Entry System

M/z- mass to charge ratio

Ppm – parts per million

RT – retention time

Min- minutes

MS/MS dot product similarity score - quantitative metric used to compare experimental mass spectra against library references
